# The neuroepithelial origin of ovarian carcinomas explained through an epithelial-mesenchymal-ectodermal transition enhanced by cisplatin

**DOI:** 10.1038/s41598-024-76984-9

**Published:** 2024-11-26

**Authors:** David Díaz-Carballo, Ayesha Safoor, Sahitya Saka, Adrien Noa-Bolaño, Flevy D’Souza, Jacqueline Klein, Ali H. Acikelli, Sascha Malak, Udo Rahner, Amin T. Turki, Anne Höppner, Annabelle Kamitz, Wanlu Song, Ye-guang Chen, Lalitha Kamada, Andrea Tannapfel, Sebastian Brinkmann, Crista Ochsenfarth, Dirk Strumberg

**Affiliations:** 1 Institute of Molecular Oncology and Experimental Therapeutics, Division of Hematology and Oncology, Ruhr University Bochum Medical School, Marien Hospital Herne, Düngelstr. 33, 44623 Herne, Germany; 2 Department of Medical Sciences, School of Veterinary Medicine, University of Wisconsin-Madison, Madison, USA; 3 The State Key Laboratory of Membrane Biology, Tsinghua-Peking Center for Life Sciences, School of Life Sciences, Tsinghua University, Beijing, 100084 China; 4 Clinic of Pediatric Oncology, Hematology and Immunology, Düsseldorf University Hospital , 40225 Düsseldorf, Germany; 5 Institute of Pathology, Ruhr University Bochum, Medical School, Bürkle-de-La-Camp-Platz 1, 44789 Bochum, Germany; 6 Department of General and Visceral Surgery, St. Josef-Hospital, Ruhr University Bochum, Medical School, Bürkle-de-La-Camp-Platz 1, 44789 Bochum, Germany; 7 Department of Anesthesia, Intensive Care, Pain and Palliative Medicine, Ruhr-University Bochum Medical School, Marien Hospital Herne, 44625 Herne, Germany

**Keywords:** Tumor cell heterogeneity, Cancer stem cells, CSC markers, Cancer, Cell biology, Stem cells

## Abstract

Acquired resistance to platinum-derived cytostatics poses major challenges in ovarian carcinoma therapy. In this work, we show a shift in the epithelial-mesenchymal transition (EMT) process towards an “ectodermal” conversion of ovarian carcinoma cells in response to cisplatin treatment, a progression we have termed epithelial-mesenchymal-ectodermal transition (EMET). EMET appears to occur via the classical EMT as judged by a) the downregulation of several epithelial markers and b) upregulation of Vimentin, accompanied by various embryonal transcription factors and, importantly, a plethora of neuronal markers, consistent with ectodermal differentiation. Moreover, we isolated cells from ovarian carcinoma cultures exhibiting a dual neural/stemness signature and multidrug resistance (MDR) phenotype. We also found that the epithelial cells differentiate from these neural/stem populations, indicating that the cell of origin in this tumor must in fact be a neural cell type with stemness features. Notably, some transcription factors like PAX6 and PAX9 were not localized in the nucleoplasm of these cells, hinting at altered nuclear permeability. In addition, the neuronal morphology was rapidly established when commercially available and primary ovarian carcinoma cells were cultured in the form of organoids. Importantly, we also identified a cell type in regular ovarian tissues, which possess similar neural/stemness features as observed in 2D or 3D cultures. The signature of this cell type is amplified in ovarian carcinoma tumors, suggesting a neuroepithelial origin of this tumor type. In conclusion, we propose that ovarian carcinomas harbor a small population of cells with an intrinsic neuronal/stemness/MDR phenotype, serving as the cradle from which ovarian carcinoma evolves.

**Supplementary Information:**

The online version contains supplementary material available at 10.1038/s41598-024-76984-9.

## Introduction

Ovarian cancer (OC) is the most lethal gynecological cancer worldwide. About 90% of all OCs are of epithelial origin, with serous subtype accounting for around 80% of cases. This type of cancer is frequently diagnosed at very advanced stages for which therapeutic options are generally not curative. The treatment of OC is based on surgery and chemotherapy with platinum- and taxane-based compounds. However, the development of resistance against platinum-based chemotherapy is a serious problem in the treatment of OCs. The intra-abdominal dissemination of OC in the form of cell aggregates in ascites or micrometastases within the peritoneal cavity, is largely driven by the epithelial-mesenchymal conversion of malignant tumor cells. Of note, the downregulation of E-Cadherin, the upregulation of N-Cadherin along with several associated proteins, appear to play paramount roles in epithelial—mesenchymal transition (EMT)^1^ making it a potentially targetable process in the treatment of OCs.

Ovarian surface cells derive from the intermediate mesoderm, specifically from the Müllerian paramesonephric ducts (after the invagination of the coelomic epithelium) whereas the fallopian tube derives from the “primary Müllerian system”^2–4^. This differential lineage is vastly distinct from the neural crest cells (NCCs), which derive from the embryonic ectoderm. Importantly, NCCs represent a unique, multipotent cell type in vertebrates which give rise to neuronal-related cell types including peripheral, enteric neurons and glial cells as well as craniofacial cartilage and bone cells, smooth muscle cells, melanocytes and, importantly, the mesenchyme^5,6^.

Epithelial-mesenchymal transition is the process by which polarized epithelial cells detach from the basal membrane upon exposure to intrinsic/extrinsic genotoxic stress and undergo epigenetic reprogramming. These changes result in the acquisition of a mesenchymal phenotype characterized by enhanced migration and invasiveness capacity, and the production of a particular extracellular matrix (ECM)^7^. It is worth mentioning that the term “mesoderm” is rather ontogenetic, whereas “mesenchyme” is histologic (embryonic connective tissue). The mesenchyme harbors multipotent cells and has the capacity to differentiate into various cell types.

EMT can be divided into three types^8,9^. Type I is the process of generating secondary mesenchymal cells needed for secondary epithelia formation. Type II is involved in wound healing, tissue regeneration, and organ fibrosis. Type III is specific for malignant cells which, depending on their clonal evolution, can spawn secondary colonies at distant sites. The reverse process, i.e. mesenchymal-epithelial transition (MET), takes place at these distant sites and, depending on the context of the new microenvironment, these cells can re-enter the EMT cycle. The process is thus very plastic, as cells with this trait can shift either to a mesenchymal or an epithelial state. Of note, cells with (also partial) EMT-MET phenotypes like cancer stem cells (CSCs) quickly adapt to changing environments because they are intrinsically refractory to chemo or radiation-based treatments^10–12^. Hence, EMT-MET-linked cell plasticity is not only confined to the ability to acquire chemoresistance or migration/invasiveness capacity, but it also involves the ability to activate dedifferentiation programs^13,14^ through which one type of cell differentiates into another, mainly orchestrated by environmental determinants. “Transdifferentiation” is hence limited to “differentiated” cells, and “metaplasia” comprises both transdifferentiation and switching in to stem cells^15,16^. However, “lineage reprogramming” could happen in a stem cell-independent way^17^. The MET-driven conversion of cells which originally belong to the same embryonal layer, regardless of the type, has been previously reported. In fact, this phenomenon has been observed in both normal and malignant cells. In the case of ectoderm-derived cells, an example that illustrates ecto-ecto conversion are nevomelanocytes. Nevomelanocytes partially recapitulate morphological and phenotypical features in the early stages of the neural crest-derived Schwann cell and Wagner-Meissner corpuscle development^18^. Interestingly, these changes do not occur in the histologically related primary invasive and metastatic melanoma. However, the expression of neuroepithelial stem cell markers like Nestin has been reported in melanomas^19^, indicating a retention of atavistic neuronal features.

Multipotent adult progenitor cells (MAPC) are not only found in bone marrow but also distributed throughout all organs. These neural stem cells can also differentiate into various hematopoietic cells^20^. It is known that mesodermal-derived cells originate outside of the nervous system, migrate into the hypothalamus where they transdifferentiate into LHRH-producing neurons^21^. Similarly, bone marrow-resident cells can migrate to the brain and differentiate into cells typically found in the CNS. These cells, such as Purkinje cells, demonstrate the capacity of mesodermal-derived cells to adopt neural cell fates^22,23^. The mechanisms behind this interconversion are still under investigation, but it is known that bone-marrow-derived cells (BMDCs) fuse spontaneously with several cell types like Purkinje neurons, cardiomyocytes and hepatocytes, creating heterokaryons^24^.

Another intriguing observation is the conversion of malignant cells after chemical insults. Some authors have described the development of malignant cells with a mesenchymal phenotype via chemically induced polyploid giant cancer cells (PGCCs). For instance, MDA-MB-231 PGCCs activate programs resembling osteogenesis, chondrogenesis and adipose differentiation after culturing in differentiation media. Notably, once xenografted, these cells were able to form tissues with full histological characteristics, indicating that these programs were stable beyond the in vitro cultures^25^. By the same token, glioblastoma multiforme cells can transdifferentiate into functional vascular endothelial cells in hypoxic conditions, which implies that cells derived from the ectoderm convert into cells derived from the mesoderm^26^. CSC phenotypes can also be induced with nitric oxide, which disrupts the Oct-4:Caveolin-1 complex^27^.

Our data support a process that describes the molecular and morphological cell identity conversion of ovarian cancer cells transforming from mesodermal to neuronal-like (ectodermal) cells driven by cisplatin exposure. We called this phenomenon “Epithelial-Mesenchymal-Ectodermal Transition” (EMET). We identified a cell type which can be assigned to the neuroepithelial class of cell. These kind of cells are rarely found in regular ovarian tissues, but hugely amplified in ovarian carcinomas, indicating a possible neuronal origin of ovarian cancers, which, according to our data, might explain why ovarian carcinomas have a strong neuronal background which can be enhanced using genotoxic agents like cisplatin.

## Methods

### Ethical considerations, patient material, animal samples and cell lines

This study was approved by the Ethics Commission of the Ruhr-University of Bochum, Medical School (register numbers: 4042-11 and 5235-15) and all methods were performed in accordance with their relevant guidelines and regulations. Written informed consent was obtained from each participant. Samples were anonymized, coded and made accessible only to research staff.

Lewis rat’s brains were obtained *post mortem* as remaining organs from an earlier study approved by the Animal Care Committee of NRW, Germany (Protocol No. 84–02.05.40.16.061). All studies were carried out and reported according to the German Animal Welfare Act and ARRIVE (Animal Research: Reporting of In Vivo Experiments) recommendations and guidelines^28^.

The non-serous SKOV3 (ATCC® HTB-77™) and the high-grade serous (HGS) OVCAR-3 (HTB-161 ™) ovarian carcinomas cell lines^29^ were obtained from the tumor bank of the University of Duisburg-Essen, Medical School. Cells were cultured in DMEM (PAN Biotech, Aidenbach Germany) containing 10% heat-inactivated foetal calf serum (FCS) supplemented with 6 µg/mL doxycycline and incubated at 37°C in a humidified atmosphere of 5% CO_2_. The OVCAR-3 cell line was cultured as described above and supplemented with 0.25 U/mL human recombinant insulin (PAN Biotech, Aidenbach Germany).

The primary ovarian carcinoma cells OC236 and OC2810 were isolated from ascites from patients and cultured in DMEM/F12 containing 10% FCS and 1% Penicillin/Streptomycin/Amphotericin B (PAN Biotech, Aidenbach Germany)^30^ All cell cultures were periodically tested for mycoplasma using MycoSPY® kit from Biontex. Normal colon patient resections were obtained from the Surgery Division of Marien Hospital Herne (Germany). Rat brain cells were immediately isolated post-mortem from brain tissues of Lewis rats obtained from the animal laboratory of Ruhr-University Bochum. Neuronal-derived cells were cultured in Neuropan Basal Medium (Pan Biotech, Aidenbach Germany), supplemented with neuronal growth factors.

### Neuron-like differentiation of SKOV3^WT^ and OVCAR-3^WT^ cells via cisplatin exposure

SKOV3^WT^ and OVCAR-3^WT^ cells were cultured in DMEM medium until the cells reached about 70% confluence. At this point medium was replaced with DMEM containing 1–2 µg/ml cisplatin (about 1 × of the IC_50_ values, see Figure SF1) and incubated until the neuronal-like appearance was observable (typically within two weeks). After this period, cells were renamed as SKOV3^CP^ and OVCAR-3^CP^ (see Figure SF2).

### Isolation of neuronal cells

Colon neuronal cells: normal colon resections of approximately 4 × 4 mm were cut from surrounding tumor areas and washed three times with pre-chilled Hank´s solution containing 1% Penicillin–Streptomycin-Amphotericin B (Pan Biotech, Aidenbach Germany), Gentamycin 25 µg/mL and Metronidazole 50 µg/mL. Cleaned tissues were transferred into a conical 50 mL reaction tube containing 25 mL of an enzymatic cocktail (1 mg/mL Collagenase I, Collagenase II and Dispase I in 1 × Hank´s solution), cut in small pieces with the aid of scissors and incubated at 37°C in an end-over-end rotator for two hours. Samples were then centrifuged at 500×g for 10 min, pellets were resuspended in 15 mL of trypsin 0.05% solution and incubated at 37 °C for 7 min. Trypsin treatment was neutralized using DMEM containing 10% FCS and again centrifuged at 500×g for 10 min. Undigested fragments were excluded using a 500-µm mesh filter, and cell pellets were washed by centrifugation three times in DMEM medium as mentioned above. Cells were resuspended in Neuronal Basal Medium supplemented with growth factors as well as antibiotics and antimycotics as mentioned above until they acquired a neuronal morphology (Figure SF3).

Brain neuronal cells: Lewis rats were acquired from the animal laboratory of Ruhr-University Bochum, Germany. Rats were narcotized in an isoflurane atmosphere and immediately euthanized by cervical dislocation. Skulls were opened with the aid of a costotome and one hemisphere was extracted, rinsed in Hank’s solution supplemented with antibiotics and antimicotics. Isolation of cells was identical to that of the colon-derived neuronal cells.

### Generation of organoids from ovarian serous adenocarcinoma cells

Organoids are self-organizing, self-renewing cellular clusters designed to mimic the tumor growth and cell heterogeneity of particular tissues by following their distinctive developmental programs. These cell clusters grow within an extracellular matrix that supports their 3D structural architecture and are cultured in optimized, tissue-specific growth media. In order to generate organoids from ovarian carcinoma cells, we followed the protocol previously described^31^ with some modifications. Initially, we cultivated OC cells for one week in hanging drops (1000 cells in 25 µL) to obtain spheroids. With this modification, we intended to stimulate the stemness in those 3D cultures. Spheroids were then enzymatically and mechanically dissociated using trypsin EDTA. Cells were embedded into the basal membrane matrix Geltrex™ (Thermo Fischer Scientific, MA, USA) placing domes of 3000 cells in 25 µL on plates and subsequently cultured in organoid media (Table ST1) supplemented with 5 ng/mL Betacellulin (a ligand of both ErbB4 and EGFR), and 0.2% human albumin. Media for this organotypic culture was replaced every three days. Within approximately one week, organoids were recovered from the domes via enzymatic dissociation using a cocktail containing 1 mg/mL of Collagenase I, Collagenase II, Dispase I and Hyaluronidase (Thermo Fischer Scientific, MA, USA) dissolved in DMEM/F12.

### Interfering Notch and MAP4K4 signaling pathways in OC cells

To study a possible interference of Notch signaling with the observed “axonal or dendritic arborization” in induced neuronal-like OC cells, we exposed SKOV3^CP^ cells to two γ-secretase inhibitors (GSIs), both indirect inhibitors of this cascade RG-4733 (1 µM, also known as RO4929097) and DAPT (5 µM), purchased from MedChemExpress (NJ, USA). Both drugs were simultaneous incubated with 2 µg/mL of cisplatin for 5 days. Notably, as for other tumor cell lines, we could not detect cytotoxic activity of these Notch inhibitors in SKOV3^WT^, SKOV3^CP^ and OVCAR-3^WT^ at these dosages levels using MTT proliferation assay (data not shown).

In addition, inhibition of MAP4K4 using the small molecule PF-06260933 (MedChemExpress, NJ, USA) enforces the neuroendocrine differentiation of cells in colonic organoids^32^. Furthermore, MAP4K4 expression positively correlates with Notch signaling and inhibiting the MAPK pathway leads to a diminished Notch response^33–35^. Therefore, we investigated the effect of PF-06260933 (1 µM) on Notch expression and the gain of neuronal features. Both SKOV3^WT^ and SKOV3^CP^ cells were exposed to single drugs or to the combinations of the MAP4K4 inhibitor (MAP4K4i) with each one of the Notch pathway inhibitors for 24h.

### Immunotechniques

Western blots, Immunocytochemistry and FACS analysis were performed using standard protocols as previously described^36^.

*Western blots*: for western blot analysis, cells in exponential growth phase were cultured in DMEM or in neurobasal induction medium for 10 days. The cells were then washed with cold PBS, trypsinised, resuspended in DMEM containing 10% FCS and centrifuged at 300×g for 5 min. Pellets were washed with cold PBS, centrifuged and lysed in RIPA buffer [150 mM NaCl, 1 mM EDTA, 1% Triton X-100, 1% sodium deoxycholate, 0.1% SDS, and 50 mM Tris–HCl pH 7.4] in the presence of a proteinase inhibitor cocktail according to the manufacturer’s instructions (Roche Diagnostics GmbH) for 30 min on ice and then centrifuged at 14 000×g and 4°C for 20 min. Supernatants were collected and protein concentration was measured by Bradford assay. Thirty micrograms of total protein were resolved by SDS-PAGE in a 4–12% gradient gel (Bio-Rad Laboratories, CA, USA) using Tris–glycine (0.025 M Tris–HCl, 0.192 M glycine pH 8.5) buffer, and transferred to 0.2 mm nitrocellulose membrane (Pierce Protein; Thermo Scientific, Inc.). Blots were blocked with 5% BSA or 10% non-fat milk resuspended in PBST (0.05% Tween-20 in 1 × PBS). Afterwards blots were washed with PBST and incubated overnight with primary antibodies resuspended in 1% BSA in PBST following the recommendations of the manufacturers (Table ST2). After incubation, blots were washed 3 × with PBST solution and incubated with a secondary antibody conjugated with HRP resuspended in 1% BSA in PBST for 2 h at room temperature. Immunoblots were washed 3 × with PBST and developed by Western Lightning Plus-ECL (Perkin Elmer) using a ChemiDoc XRS + system with Image Lab Version 2.0.1 software (Bio-Rad Laboratories).

*Immunocytochemistry (ICC):* cells were grown in chamber slides to appropriate densities, washed with 1 × PBS, fixed with 4% formaldehyde in PBS for 20 min, rinsed twice with 1 × PBS for 5 min, and blocked with 5% BSA (AbD Serotec, London, UK) in PBST at room temperature for 60 min. Wells were incubated with primary antibodies (Table ST2) resuspended in 1% BSA in PBST at concentrations following the manufacturers’ recommendations at 4 °C overnight. Chambers were washed 3 × with PBST and finally incubated with the appropriate secondary antibodies resuspended in 1% BSA in PBST at room temperature for 2 h in the dark. Chambers were washed once with PBST for 5 min and were stained for 15 min with 1 μg/ml Hoechst 33,258 diluted in PBST in order to visualize the nuclei. Wells were rinsed 3 × with PBST and mounted using Faramount Mounting medium (Agilent Technologies, CA, USA) for visualization in an Eclipse i-50 microscope (Nikon, Tokyo, Japan), using the NIS-Advanced imaging software.

*Immunohistochemistry (IHC):* paraffin tissue sections of 4-μm thickness were baked overnight at 60 °C to firmly attach the sections to the slides. After baking, the sections were deparaffinized in two changes of xylene-substitute (Thermo Scientific, London, UK) solution for 10–15 min and rehydrated in a series of graded ethanol solutions (100%, 100%, 95%, 70%, 50%) for 3 min each. Samples were subjected to antigen retrieval by heating the sections for 30 min in 10 mM sodium citrate buffer (pH 9.0) at 95 °C in a domestic vegetable steamer. The slides were washed twice in 1 × PBS for 5 min and blocked for 60 min with 10% normal goat serum at room temperature. Primary antibodies were applied overnight at 4 °C according to the manufacturers’ recommendations by diluting in 1% goat serum in PBST. On the next day, the slides were washed three times in PBST for 5 min each. Tissue sections were incubated with conjugated secondary antibodies (Cell Signaling, Cambridge, UK) diluted in 2.5% goat serum in PBST for 120 min at room temperature according to the manufacturers’ recommendations. Next, the samples were stained for 15 min with 1 μg/ml Hoechst 33,258 diluted in PBST in order to visualize the nuclei. The slides were then washed three times in PBST for 5 min each and rinsed in 1 × PBS for another 5 min. Tissue specimens were mounted using Faramount Mounting medium (Agilent Technologies, CA, USA) for visualization in an Eclipse i-50 microscope (Nikon, Tokyo, Japan).

*FACS analysis*: for cytometric analysis, cells were harvested and fixed with methanol/acetone (1:1) for 15 min at 4 °C and blocked with 5% BSA in PBST for 30 min. Fixed cells were centrifuged at 500×g for 5 min and pellets were rinsed three times with PBST and incubated with primary antibodies (Table ST2) diluted in 1% BSA in PBST for 2 h at room temperature according to manufacturer’s recommendations. Cells were washed three times with PBST, and incubated with secondary antibodies diluted at 1:1000 in 1% BSA in PBST for 1 h. Conjugated antibodies were used according to manufacturer’s recommendations. Appropriate isotype antibodies were used for cytometric analysis. Labelled cells were cytometrically measured using a CytoFLEX Research Cytometer B5-R5-V5 (Beckman Coulter, Krefeld, Germany). At least 2 × 10^4^ events were acquired in each measurement to account for sufficient cell number for analysis. The main population of cells was gated according to the FSC/SSC dot plot density, discriminating them from dead cells. Signals of isotype controls were used to set the gate for negative signals.

### Histochemistry

Fontana-Masson staining for the histological detection of melanin was performed using a validated kit purchased from ScyTek (Utah, USA), following their instructions. After the deparaffination of sections, slides were placed into a cuvette containing silver nitrate solution previously equilibrated with ammonium hydroxide and incubated at 58°C until tissue section became yellowish/brown. After 30 min, slides were rinsed three times in distilled water and incubated in gold chloride solution (0.2%) for 30 s. After threefold rinse with distilled water, sections were incubated with Sodium Thiosulfate Solution (5%) for 2 min and again washed in distilled water, stained with Fast Red solution for 5 min and finally covered using Eukitt® mounting resin.

### Total RNA Isolation and RT-qPCR

Cells were cultured in DMEM with 10% FCS and harvested at 70% confluency. RNA was extracted with Trizol® (Life Technologies, California, USA), treated with 7 Kunitz units of DNase I (Qiagen, Hilden, Germany) and further purified on RNeasy mini columns (Qiagen, Hilden, Germany) according to the manufacturer’s instructions. RNA integrity was ascertained using the 2100 Bioanalyzer (Agilent, Santa Clara, CA). For amplification and detection of neuronal markers, we designed specific primers and probes (Table ST4) which were synthesised by IDT Inc. (Iowa, USA). The amplification of 100 ng of cDNA was performed in triplicate with 250 nmol primers in PrimeTime® Gene Expression Master Mix (IDT, Iowa, USA) on a CFX96TM Real-Time System (Biorad Laboratories, CA, USA). Relative expression was determined using the ΔCt comparative method. Although several neuronal-related genes were detectable using qPCR, we included in our analysis only those with expression values below a ΔCt threshold of 29 cycles.

### Transcriptome analysis

0.5 µg of total RNA isolated from SKOV3^WT^ and SKOV3 exposed to cisplatin for 72 h were subjected to sequencing using Illumina next-generation sequencing (NGS) system (BGI Genomics, Hong Kong, China). The transcriptome results were analysed using different bioinformatic tools like: Dr. Tom, the Ensembl BioMart tool and gene expression clustering analysis were performed using the Enrichr platform. In addition, the genomic positions of all peaks identified before and after the treatment of SKOV3^WT^ cells with cisplatin have been intersected with the proviral coordinates of a collection of about 3280 individual HERV loci integrated in human genome, which have been characterised in detail in previous works, through the Bedtools “intersect” option. The same method was used to identify differential peaks (diffpeaks) co-localised with HERV sequences^36^.

### Single cell sorting via micromanipulator

SKOV3^WT^ and SKOV3^CP^ were cultured on a petri dish until they reached a confluency of 50%. Before the single cell sorting, the medium was replaced with fresh medium to remove floating particles or dead cells. For the single cell sorting, the TransferMan®4r Micromanipulator from Eppendorf was used. First, a cell of interest was isolated from adjacent cells, by physically removing surrounding cells with a TransferTip® (Eppendorf) glass capillary. Then, another glass capillary (VacuTip II) was used to release trypsin–EDTA solution within the vicinity of the cell of interest. After brief incubation, the cell was picked using the same VacuTip II capillary. The uptake and release of liquid into the capillary was controlled with the CellTram® 4m Oil Manual Hydraulic Microinjector. The cell was then transferred into a microcentrifuge tube, and stored on ice. This process was repeated until approximately 20 cells were picked. The picked cells were then immediately used for single cell RNA isolation.

### Single-cell RNA isolation and cDNA amplification and analysis via real-time PCR

The REPLI-g WTA single cell kit from QIAGEN was used to generate and amplify cDNA from the handpicked single cells. All steps were carried out in accordance with the manufacturer´s instructions and the incubations were performed in a Mastercycler gradient (Eppendorf). First, the picked cells were lysed for 5 min at room temperature. Then gDNA was removed prior to the WTA process. Transcripts were amplified using random oligo-dT primers. The synthesized cDNA was ligated and then amplified by MDA technology, using the REPLI-g SensiPhi DNA polymerase, in an isothermal reaction for 2 h. The amplified cDNA was quantified with a Pico Green dsDNA reagent kit from Invitrogen. The cDNA was employed for qPCR using 100 ng per reaction. Thereafter, each sample was amplified in triplicate in a CFX96 Real-Time System (Biorad Laboratories).

The expression of neuronal markers was measured by qPCR using validated primers and probes from Integrated DNA Technologies Inc. Sequence and polarity of the primers are shown in Supplemental (TableST4).

### Enrichment of EpCAM negative cells by immunomagnetic MicroBeads isolation

OC236 cells revealed a subpopulation of cells with a nested morphology, which are EpCAM negative but positive for a plethora of neuronal markers. In order to isolate such cells, we employed MicroBeads directly conjugated to EpCAM antibody (Cat. # 130–061-101, Miltenyi Biotec, Bergisch Gladbach, Germany). Briefly, cells were counted and resuspended in a separation buffer (PBS, pH 7.2, 0.5% BSA, and 2 mM EDTA), and incubated with the appropriate amount of FcR Blocking Reagent (Cat. # 130–059-901 Miltenyi Biotec) and CD326 (EpCAM) MicroBeads for 30 min at 4°C and then separated using MS MACS® Columns and a MiniMACS™ Separator. The eluted fraction was subjected to a second round of separation and positive and negative fractions were collected. The purity of these fractions was evaluated by flow cytometry.

### Statistics and reproducibility

Intergroup comparisons of medians were performed with paired Student’s t test, two-tailed. Significance was defined as p < 0.05; statistical analysis was done with OriginLab2022, v9.9.0.225 (OriginLab Corp., MA, USA). Western blots were subjected to densitometric analysis. ICC, IHC and organoid pictures were descriptive and therefore no statistical analysis was applied. All results shown are representative of at least three independent experiments.

## Results

### Cisplatin drives morphological remodeling of SKOV3 ovarian carcinoma cells into neuron-like cells

Morphological remodeling of cancer cells upon cytostatic treatments is observed in various tumors of different histology. Usually, phenotype dedifferentiation in malignant cells is attributed to processes, such as EMT, which greatly contribute to clonal evolution and tumor cell heterogeneity.

Mounting evidence suggests that malignant cells influence the tumor microenvironment (TME) by releasing a plethora of neurotrophic factors, e.g. NGF, BDNF, GDNF, Neuturin and Ephrin B1 to promote axonogenesis, neurogenesis and neural reprogramming^37^. However, little is known about the ability of some cytostatics to convert certain cancer cells into neuron-like cells which may explain the increase in neurotrophic factors in the TME.

**Fig. 1:**
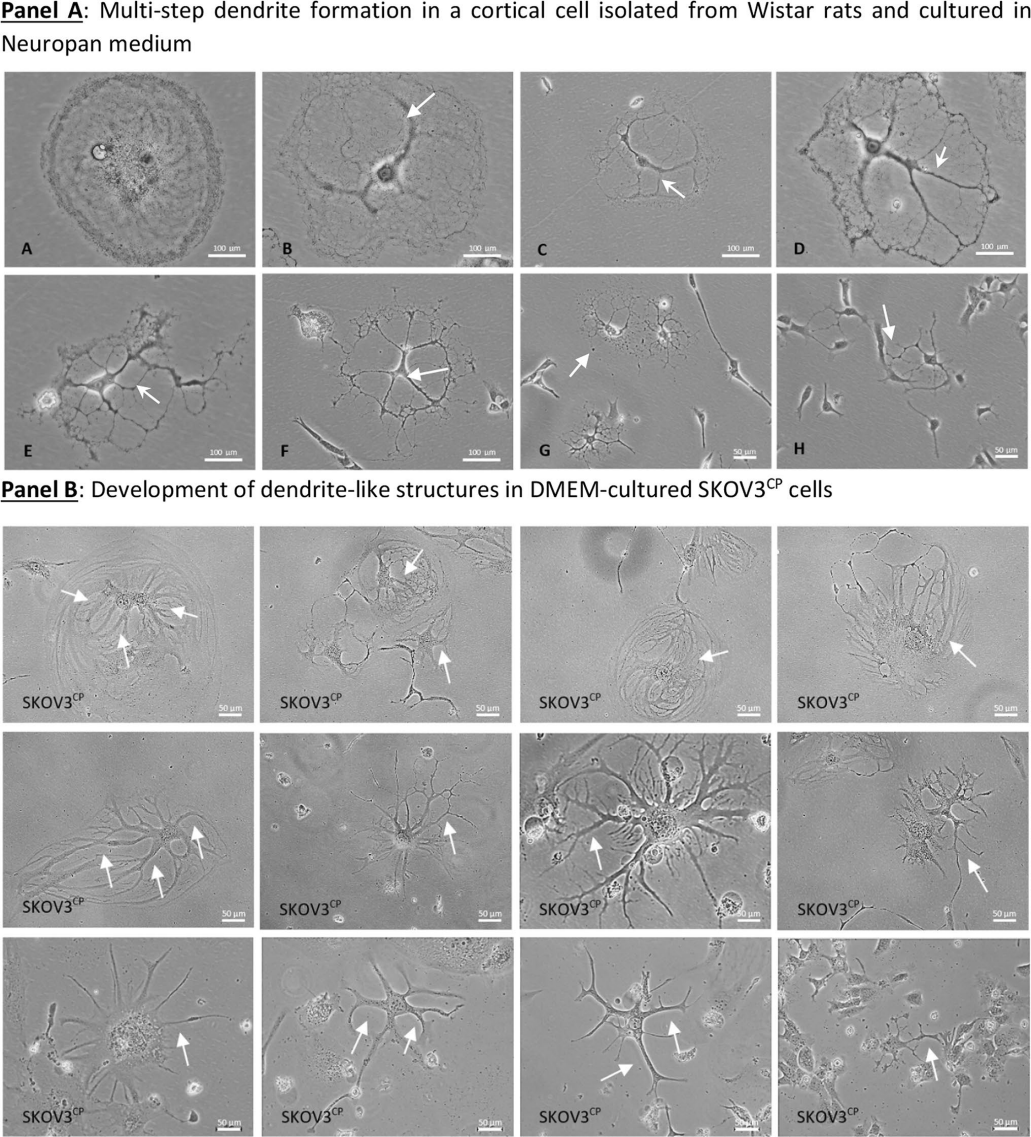
Morphological remodeling of cells with neuron-forming ability. **Panel A**: progressive cytoplasm shrinkage in rat brain cells in vitro with formation of dendritic trees, typical of multipolar neurons. The cells were cultivated in Neuropan medium after the enzymatic isolation from rat brains. A population of giant, mono- or binucleated cells (**A**) undergoing progressive cytoplasmic/cytoskeletal structural changes (**A**-**H**) and forming “branches” (white arrows, **B**-**D**) which ultimately give the cells a multipolar neuron-like appearance. **Panel B**: cellular remodeling towards neuronal morphologies of SKOV3 cells treated with sub-cytotoxic concentrations of platinum- derived drugs. The formation of neuronal structures reminiscent of multipolar neurons is clearly analogous to normal neuron development observed in brain cells isolated from rats (**Panel A**). Pictures are representative of several independent experiments.

Rat brain cell cultures grown in 2D Neuropan induction medium allow the growth and differentiation of giant cells that develop into multipolar neuron-like cells. Figure 1, Panel A illustrates the formation of dendritic trees via cytoplasm shrinkage (pictures A-D, white arrows). In a similar way, cisplatin induces a morphological conversion of ovarian carcinoma cells into neuron-like cells as observed in normal rat cells isolated from the forebrain as well as cells isolated from colon (Fig. 1, Panel B). This effect is independent of the type of medium employed, indicating that plays a key role in this process.

### Ovarian carcinoma cells with induced neuronal morphology form cell–cell communication networks

**Fig. 2:**
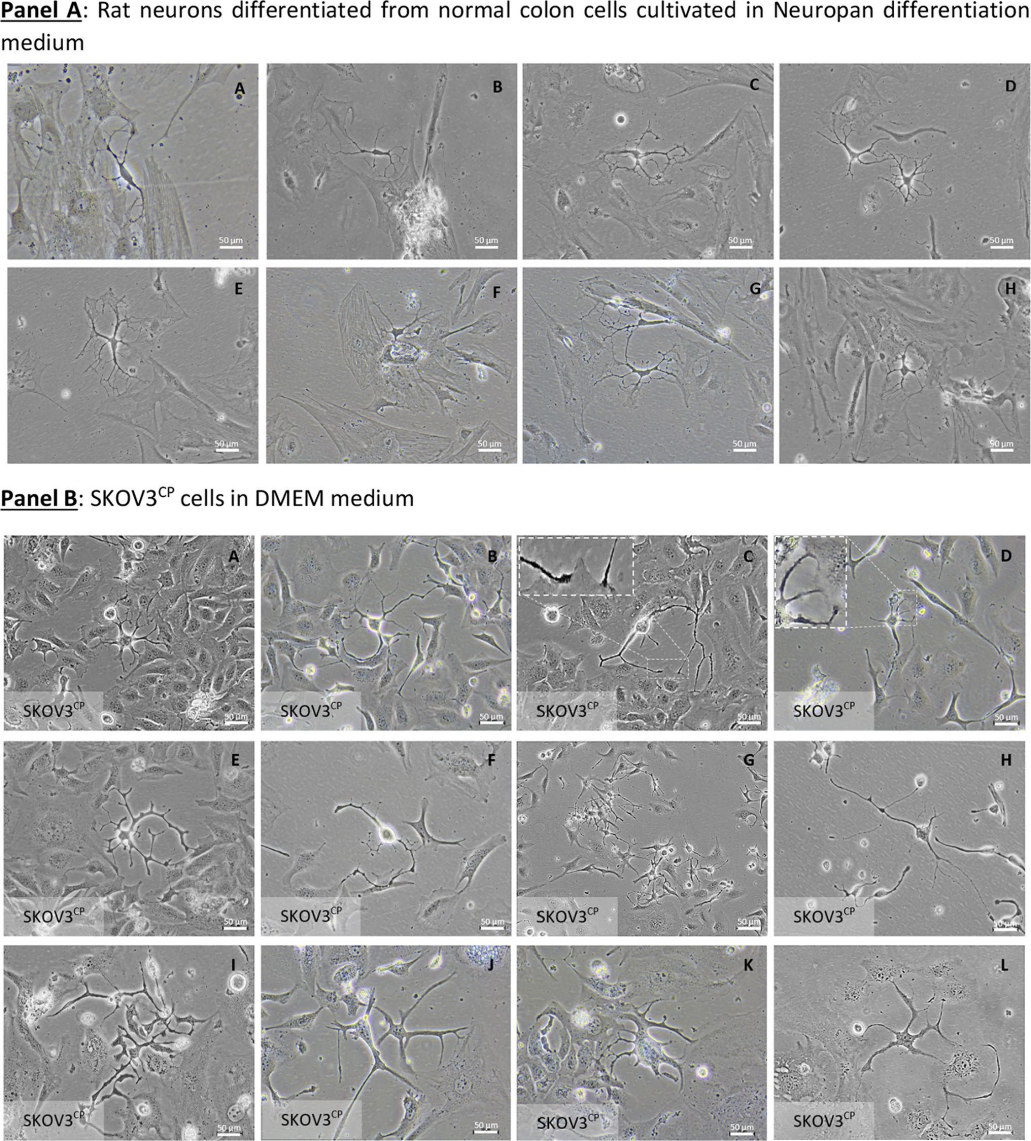

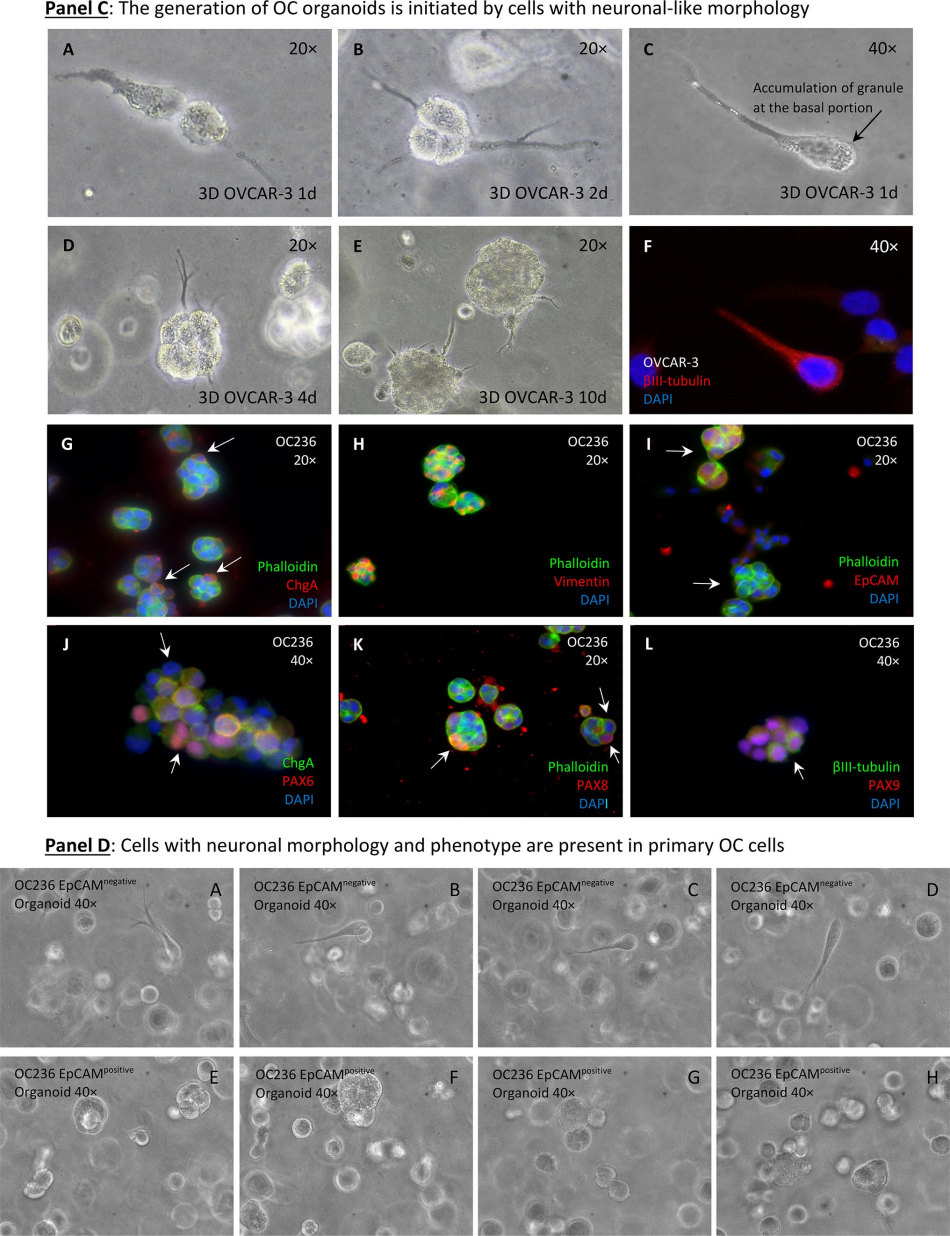
Communication networks and differentiation of cells in 2D and 3D cell cultures. **Panel A**: normal enteric neurons isolated from Wistar rats interacting with adjacent cells via neuron-somatic cell (**A**, **B**, **C**, **E**, **F** & **H**) or neuron-neuron (**D** & **G**) communication, with the contact being mediated by dendrite-like structures. **Panel B**: cell–cell communication among SKOV3^CP^ cells similar to normal tissue (neuron-like somatic cells: close-up from pictures** C** & **D**; neuron-neuron,** H**) via dendrite-like protoplasmic extensions. **Panel C**: culture of primary OC cells in 3D Geltrex gel supplemented with organoid medium. The clusters of OC cells maintained in organoid conditions revealed that in the early stages, these cells develop a neuronal appearance similar to 2D models (**A**-**E**). In this context, we observed cells with a morphology resembling colonic neuroendocrine cells of the crypts characterized by the typical basal granule accumulation (**C**). The neuronal nature of these cells is confirmed by the expression of βIII-tubulin (**F**). In 3D cultures, we observed the recapitulation of ChgA expressing cells (**G**, white arrows). Vimentin was widely expressed in these cultures (**H**), while EpCAM was differentially expressed among cell clusters (**I**, white arrows). The transcription factors PAX6, PAX8 and PAX9 were differentially expressed by cells within the organoids (**J**-**L**, white arrows). **Panel D**: Primary ovarian carcinoma 3D cell cultures with neuronal morphology and phenotype. OC236 subpopulations grow in nest-like forms with EpCAM^negative^ cells sorted from the EpCAM^positive^ fraction using immunomagnetic MicroBeads. In organoid cultures derived from EpCAM^negative^ fraction, cells with a neuronal phenotype, similar to those observed in 2D cell cultures (**Panel C**, **A**-**C**) appeared. Contrarily, in EpCAM^positive^ cells this phenotype is rare to find (**Panel D**, **E**–**H**). Of note is the tendency of EpCAM^positive^ cells to generate clusters similar to organoids. Pictures are representative of several experiments. Magnification is reflected in each picture.

Communication networks between neurons and other cell types in 2D-cultured normal rat enteric cells are illustrated in Fig. 2, Panel A showcasing interactions between neuronal and somatic cells. SKOV3 cells after exposure to cisplatin undergo a neuronal de-differentiation and initiate a prominent network with adjacent cells or with other neuronal like cells (Fig. 2, Panel B) similar to the cell–cell interactions observed in cultures of differentiated neurons isolated from normal colon. Neuron-like cells attach to somatic cells and establish communication through cellular protrusions resembling those found in neuromuscular junctions (Fig. 2, Panel B close-up pictures C & D and Videography A).

### Generation of OC organoids initiated by cells with neuronal-like morphology

In 2D cultures of OC cell lines, a small population of cells with a neuronal-like morphology is recurrently observed, as previously demonstrated. Contrarily, during the organoid differentiation, particularly within the first two days of culture, a prominent neuronal-like population is observed (Fig. 2, Panel C, A-E & SF4). It is known that the formation of organoids in 3D models typically originates from stem cells^38^. Consequently, those cells with an initial neuronal-like appearance from which an organoid arise, are the progenitor cells. This suggests that organoid differentiation, including the development of cell heterogeneity, is driven by these neuronal-like cells, which initially resemble neuroendocrine cells of the colonic crypts. Regarding the morphology of OC organoids, it reflects the phases of cell cluster formation observed directly in freshly collected ascites, where a cystic formation differentiates to solid cell clusters (see Videography B).

In the Fig. 2, Panel C, we observed the cell heterogeneity recapitulation in organoids generated from primary ovarian carcinoma cells, isolated from ascites. Cells, expressing high amounts of Chromogranin A (ChgA) were detected (G, white arrows). Notably, many cells within the organoids express basal levels of ChgA. The expression of Vimentin, a type III intermediate filament expressed in mesenchymal cells, was observed in all organoids scrutinized (H), indicating an EMT process in those entities. Contrarily, EpCAM was differentially expressed among organoids (I, white arrows) revealing the different fates of cells in distinct clusters. The expression of PAX transcription factors was observed in a subset of cells within the cellular clusters (J-L, white arrows).

### Acquisition of neuronal morphology by ovarian carcinoma cells begins with the propagation of a distinctive cell type

**Fig. 3:**
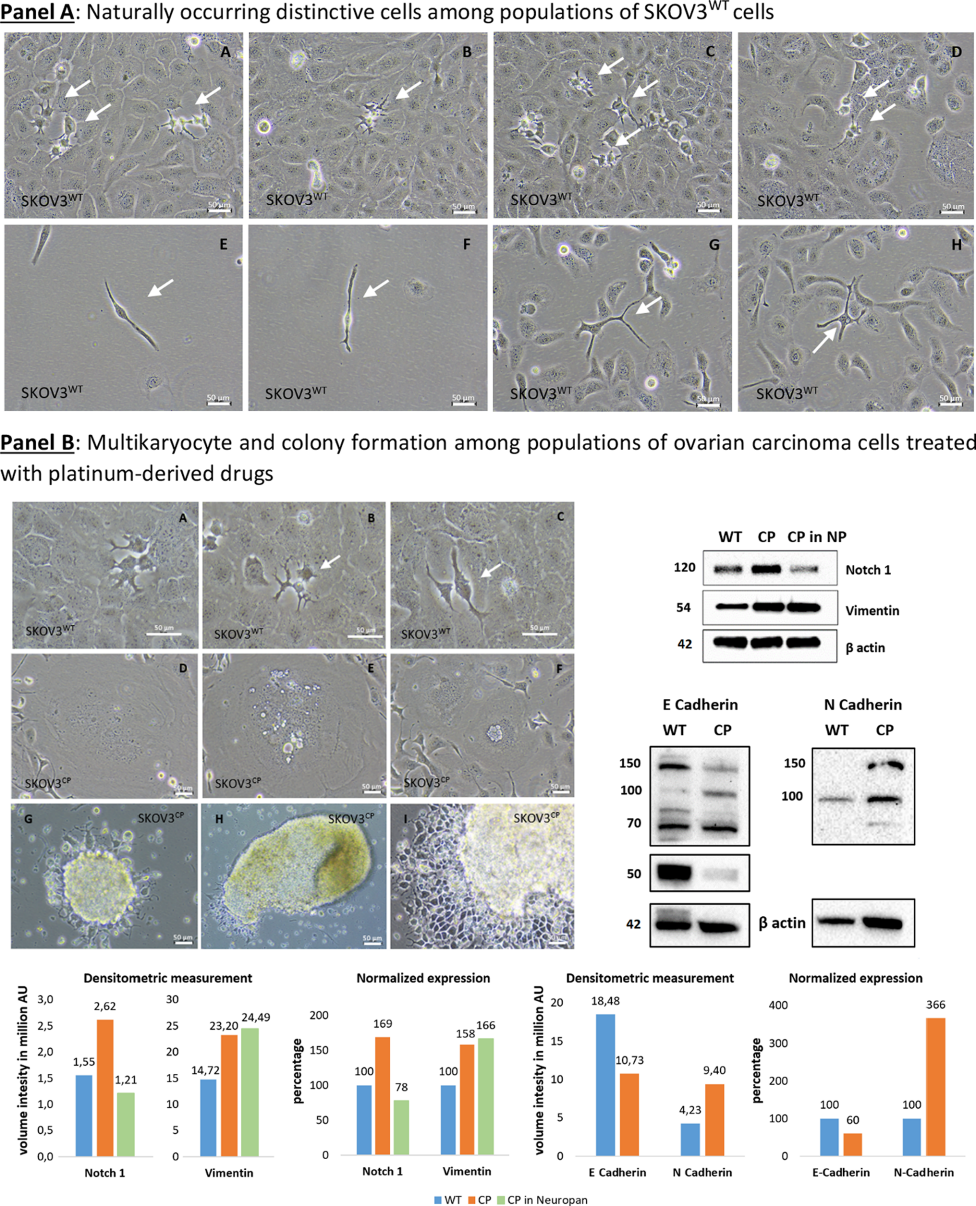

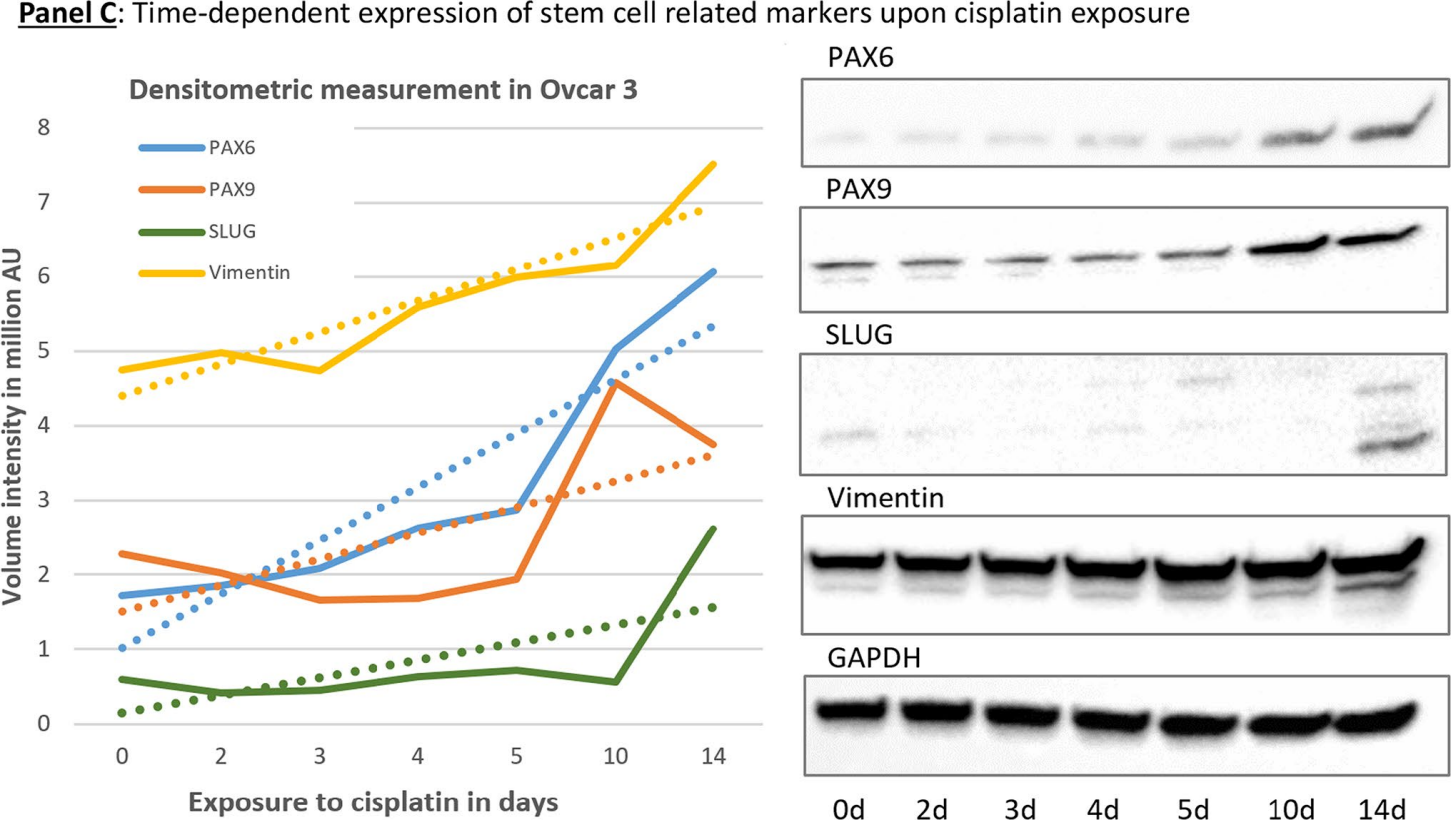
Cells with the capacity to convert into neuron-like cells among SKOV3 cell populations and time-dependent changes in gene expression. **Panel A**: naturally occurring, dark-colored cells generating cells with different morphology than the whole cell population (arrows in pictures **A**-**D**). Another cell type are cells with two or multiple protrusions (arrows in pictures **E**–**H**). **Panel B**: changes in SKOV3 cell morphology upon platinum-derived drug exposure. Naturally occurring cells with distinctive morphology in SKOV3^WT^ cells (**A**-**C**). Multikaryocyte formation (**D**-**F**) was a frequent event in SKOV3 cells treated with cisplatin. SKOV3^CP^ cell populations cultured in 2D Neuropan neuronal induction medium form typical iPSC-like colonies which generate cells which are morphologically clearly different from the parental SKOV3 cell population (**G**-**I**). The expression of Notch1, E-cadherin and Vimentin was studied by Western blot, revealing that EMT takes place in SKOV3^CP^ cells. Lane 1, SKOV3^WT^ cells cultured in 2D DMEM; lane 2, SKOV3^CP^ cells in 2D DMEM; lane 3, SKOV3^CP^ cells in 2D Neuropan neuronal induction medium. Interestingly, Notch1 was downregulated in Neuropan-cultured cells. We named the cells having a different morphology than the rest in wildtype populations as “twin” cells (**Panel A**, **A**-**D**; **Panel B**, **A**-**C**). **Panel C**: Inducible expression of stem cell related markers upon cisplatin exposure. The time-dependent expression of the transcription factors PAX6, PAX9 and Slug, as well as the mesenchymal marker Vimentin were monitored by immunoblotting. Despite of the oscillatory expression of those markers upon cisplatin exposure, the trend observed in the expression kinetics clearly revealed an accumulation of those markers in treated cells. This effect was accompanied with morphological changes towards a “neuronal” shape. Values represent the expression of such proteins relative to GAPDH after densitometric analysis. **AU**: arbitrary units Representative experiments for n = 3.

Microscopic inspection of SKOV3^WT^ cells reveals a naturally occurring, distinctive cell type with a morphology resembling that of bipolar and multipolar neurons. These cells are clearly different in shape and color from the rest of the population and attach to adjacent cells during cytokinesis (Fig. 3, Panel A, white arrows in pictures A-D or Panel B, pictures A-C). We called these cells “twin” because they become more prominent during cell division, and analyzed their transcriptional signature for stemness and neuronal markers (see Fig. 6 and Videography B & C). Interestingly, we found similar cell types in primary cultures of ovarian carcinomas obtained from ascites (Figure SF5 & SF6).

SKOV3^CP^ cell subpopulations undergo karyokinesis without cytokinesis, resulting in the formation of giant multikaryocytes (Fig. 3, Panel B pictures D-F) which, via cellularization, lead to an increase of colonies once platinum-based drugs are eliminated from the medium. These events were reported by our group and others^39^. Incubating SKOV3^CP^ cells in neuronal induction medium drives the formation of colonies very similar to the ones obtained by induction of pluripotent cells (Fig. 3, Panel B pictures G-I).

Western blot analysis of the stemness features in cells treated with cisplatin and maintained in normal DMEM medium revealed that, SKOV3^CP^ cells upregulate Notch1, Vimentin and N-Cadherin while downregulating the epithelial protein E-Cadherin, compared to SKOV3^WT^ cells (Western blots from Fig. 3, Panel B), indicating an EMT process. This effect is also partially observed in cells treated with cisplatin maintained in Neuropan induction medium, though notably Notch1 expression is significantly reduced. However, detecting E-Cadherin with commercial antibodies often reveals bands at different molecular weights, depending on the cell type and its physiological state^40,41^.

### Stem cell related-markers are accumulated during cisplatin exposure

Transcription factors (TFs) often function synergistically to regulate gene sets essential for specific biological processes. In the OVCAR-3 ovarian carcinoma cell line, we observed a time-dependent increase in certain proteins that are key regulators or indicators of stem cell processes. OVCAR-3 cells exposed to cisplatin for 14 days revealed profound changes in the cell morphology towards “neuronal” phenotype. These changes were primarily accompanied by the time-dependent accumulation of some TFs such as PAX6, PAX9 and Slug, however, are probably supported by EMT as indicated by the accumulation of Vimentin (Fig. 3, Panel C). Nevertheless, in other ovarian carcinoma cell types, the kinetics of such markers oscillated. Notably, PAX6 accumulation was consistent across different cell lines.

### Epithelial-neuronal gene expression and phenotypic switch in SKOV3 cells induced by cisplatin

To determine the molecular basis of the morphological conversion of epithelial cancer cells into neuron-like cells under the genotoxic stress of cisplatin, we first conducted a gene expression analysis focusing on markers indicative of neuronal conversion.

**Fig. 4:**
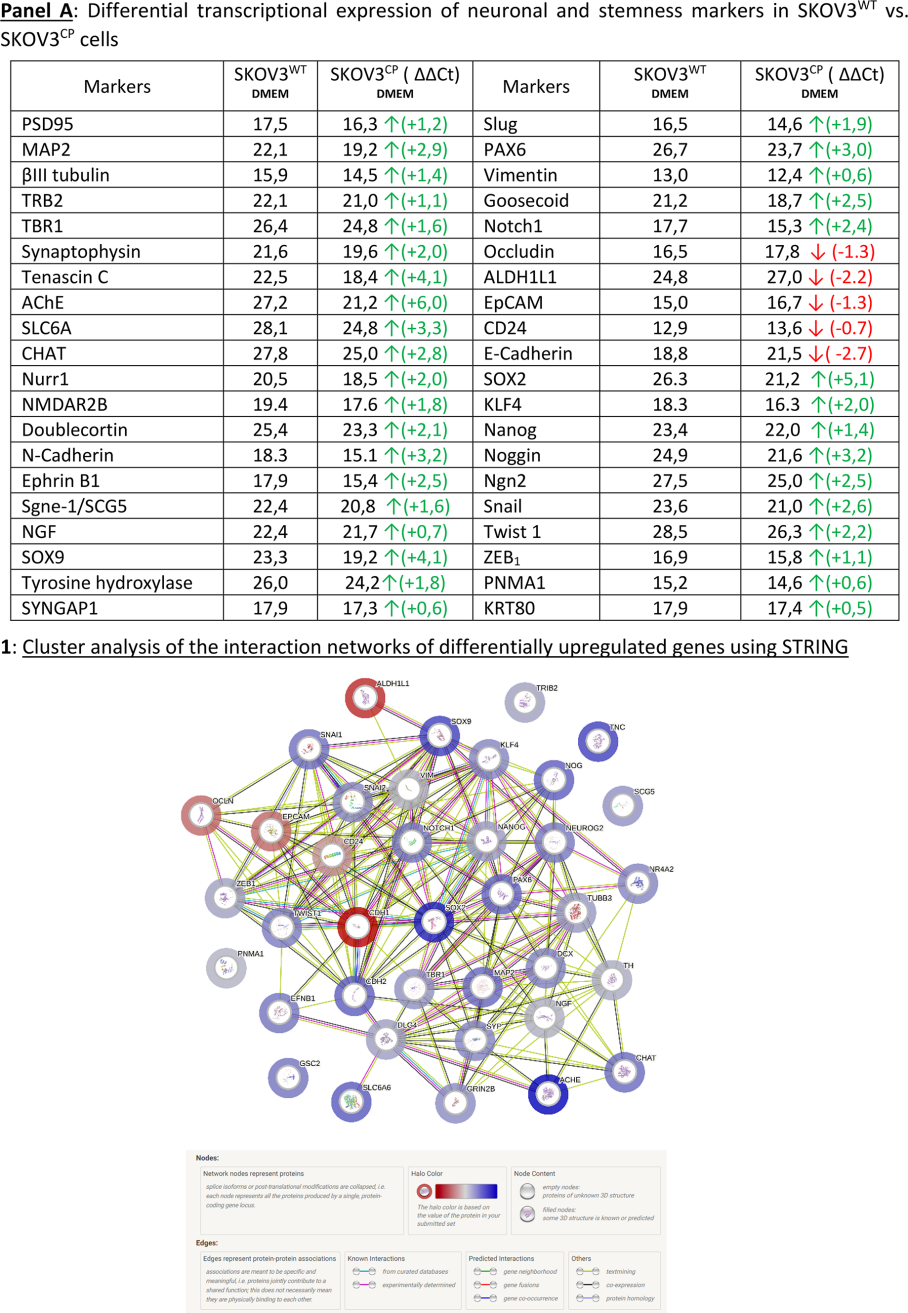

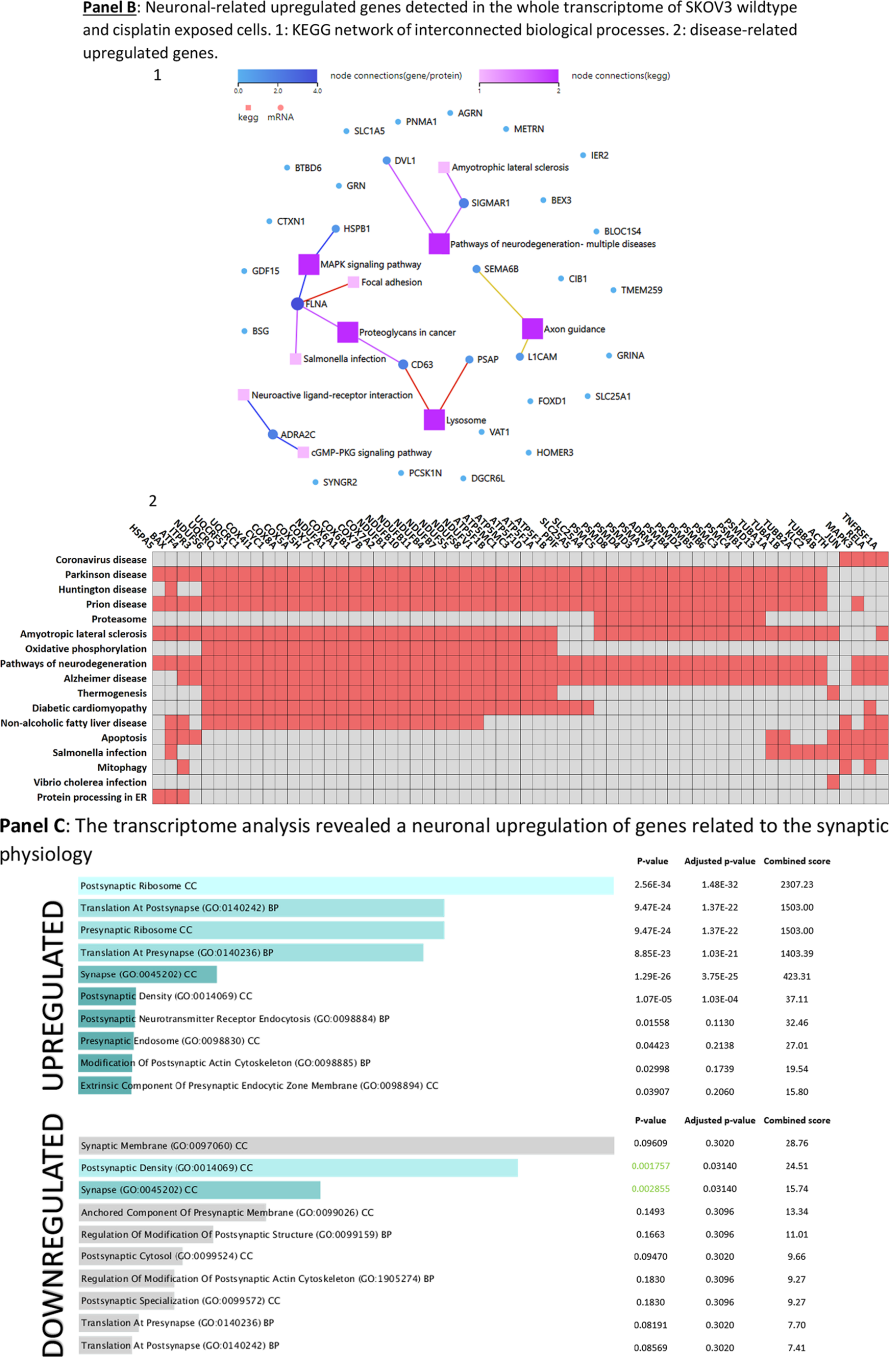
Expression of EMT-related proteins in SKOV3^CP^. **Panel A**: ΔΔCt values for transcription factors and neuronal markers (RT-qPCR TaqMan system). Transcriptional signatures defining a neuronal phenotype are clearly demonstrated in SKOV3^CP^ cells. Of note, epithelial markers like Occludin, ALDH1L1, EpCAM, CD24 and E-cadherin were significantly downregulated. Green arrows denote an upregulation, red arrows a downregulation at the transcriptional level. Data represents only those genes which were differentially expressed below the ΔCt threshold of 29 cycles. **A1**: Cluster analysis of the interaction networks of differentially expressed genes using STRING (http://www.string-db.org) with default parameters, including a minimum interaction score of 0.4 (medium confidence) and various interaction sources including Text mining, Experiments, Databases, Co-expression, Neighborhood, Gene Fusion, and Co-occurrence. The colored “halo” around the bubbles indicates differential expression values, with red indicating higher values in SKOV3^WT^ and blue indicating higher values in SKOV3^CP^. **Panel B**: **1**, Transcriptome analysis of neuronal-related differentially expressed genes in SKOV3^WT^ vs. SKOV3^CP^ cells using NGS. SKOV3 cells were exposed to cisplatin for 72 h to study the first biological response to this agent. We extracted from the transcriptome results the neuronal-related genes found enhanced in SKOV3 cells treated with cisplatin for 72 h and depicted the existence or until unknown interconnection of more than 32 different genes related to neuronal processes found upregulated in cisplatin exposured cells. One relevant gene, which has not escaped our attention, was PNMA1 that codes the paraneoplastic Ma1 antigen a truly specific neuronal protein. **2**, Gene expression clustering analysis using the SynGO 2022 database (https://maayanlab.cloud/Enrichr/) revealing the upregulation of several genes related to neurodegenerative diseases and neuronal physiology. **Panel C**: Gene expression analysis using the SynGO 2022 database from the Enrichr online platform revealed that there are significant enrichments of gene sets associated with synaptic function.

Figure 4, Panel A reflects the data gathered by qPCR with a TaqMan system. We inferred from the genetic expression analysis that the cells, at the population level, had lost their epithelial state, as judged by the downregulation of key epithelial markers like E-Cadherin, EpCAM, Occludin, CD24, and ALDH1L1. Concurrently, transcription factors associated with stemness and EMT like Slug, SOX2, Klf-4, Notch1 and Nanog were clearly upregulated, as was Vimentin. Of particular note is the upregulation of Tribbles-1 and 2 (TRB 1 & 2), which act as tumor suppressors. Occludin, a NADH oxidase typically localized at tight junctions in epithelial cells, plays crucial roles in tight junction assembly and barrier maintenance. However, in cancer cells, high levels of Occludin are associated with reduced invasiveness and motility. Therefore, its downregulation by cisplatin in ovarian carcinoma cells may promote migration and invasion. This downregulation aligns with the loss of epithelial traits and the acquisition of mesenchymal features (Fig. 4, A). It appears that the exposure to cisplatin enhances the transcription factor Neurogenin 2 (Ngn2), which is involved in the development of motor neurons (Fig. 4, A).

The transcription of Snail (SNAI1) was upregulated upon cisplatin exposure. Snail, similar to Slug (SNAI2), is a transcriptional repressor of ectodermal genes within the mesoderm and also represses E-Cadherin. Its upregulation contributes to EMT, inducing a downregulation of some ectodermal genes, which were upregulated after cisplatin exposure (Fig. 4, A). In summary, the cells responded to changes in ectodermal gene expression, and the activation of the Snail pathway served as a mechanism to restore homeostasis.

Another relevant finding is the upregulation of typical neuronal markers involved in neurogenesis like PAX6, along with structural proteins like βIII-tubulin and MAP2, which are almost exclusively found in neurons. Tenascin C, a protein mostly restricted to the extracellular matrix (ECM) in neurogenic domains of the nervous system was also upregulated. Moreover, certain key players in the physiology of neuronal transmission such as synaptophysin and ACHE (acetylcholinesterase), or the neuronal receptor NMDAR2B and the nuclear receptor 4A2 (NR4A2) or Nurr1 showed increased expression (Fig. 4, A).

The protein association network derived from the transcriptional data revealed an interconnected system, where proteins such as Tenascin, SNURF, and SCG5 appeared to be not functionally related to the majority of other proteins (Fig. 4, Panel A1). It is worth to mention that this network was created considering pre-existing data. Consequently, we identified proteins involved in the global transcriptional regulation induced by cisplatin in the SKOV3 ovarian carcinoma cell line. The extensive expression of genes associated with neuronal systems, along with their physiological characteristics, strongly suggests a neuronal background for these cells. Notably, the presence of the paraneoplastic Ma antigen 1 (PNMA1), which is specific for neurons, provides clear evidence of the neuronal origin of these cells. Figure 4, Panel B1 depicts the KEGG interconnection of neuron-related genes, extracted from transcriptome analysis performed in SKOV3^WT^ and SKOV3^CP^. It is worth mentioning is that this analysis of the whole genome was performed after 72 h of cisplatin exposure. Of note, PNMA1 is present in both SKOV3^WT^ and SKOV3^CP^ ovarian carcinoma cells and was enhanced in cisplatin-exposed cells, as corroborated using different methods (Fig. 4, Panel A; Fig. 7, Panel A, K & O). This is clearly indicative of the neural ontogeny of this cell line. Moreover, other genes related to the neuromuscular junction like the MuSK activator Agrin, Membralin and SLC25A1 are expressed in SKOV3^WT^ ovarian carcinoma cells and overexpressed in SKOV3 cells exposed to cisplatin. We have to keep in mind that in the transcriptional study conducted by qPCR, the transcription factor Neurogenin 2, which is implied in the development of motor neurons, is also upregulated upon cisplatin exposure. In this context, it is noteworthy that the transcriptome analysis of both wildtype and cisplatin-exposed cells revealed the expression of numerous genes associated with the muscle system and morphogenesis. (see Figure SF7).

Figure 4, Panel B2, illustrates a significant multitude of genes associated with various diseases, notably showcasing a prevalence of genes linked to degenerative or neurological conditions characterized by impaired movements, including Parkinson’s, Huntington’s, Amyotrophic lateral sclerosis (ALS), Alzheimer’s, and Bovine spongiform encephalopathy (BSE).

We observed in 2D & 3D cultures of OC cells, the tendency of cells to build communication networks similar to those formed between neurons (Fig. 2) or neurons and other cell types. In fact, some genes related to synaptic physiology e.g. Synaptophysin (Fig. 4, Panel A) were detected in cells of this cancer entity. The analysis of the transcriptome revealed the expression of genes related to neuronal synapsis (Fig. 4, Panel C). However, electrophysiological studies revealed no electrical activity in such cells and therefore the dendritic-like structures (data not shown) remain dysfunctional. This could be based on the imbalance of up- and down-regulated genes, which form a proper functional synapsis.

In all of the transcriptome data resulting from NGS, we decided to search for the fate of the HERV expression, given the fact that in resistant cancer cells the expression of envelope proteins mediate cell–cell fusion, a phenomenon often observed in ovarian carcinoma cells (see Videography E). This process is thought to be involved in metastization events. Ovarian carcinoma cells express a plethora of endogenous retrovirus elements. Using qPCR, we validated the differential expression of HERV elements in SKOV3 wildtype and cisplatin resistant cells (Figure SF8, I, II & III). It is worth mentioning that we globally observed a downregulation of several HERV elements in SKOV3^CP^ compared to its wildtype. However, we detected some upregulated HERV genes, which code for envelope proteins like, HERV-W_E1_ (Syncytin 1), HERV-FRD_1_ (Syncytin 2) and HERV-W_env_, which have retained their fusogenic properties. These proteins have been widely reported to be highly fusogenic in addition to their elucidated involvement in immune escape, which is mediated by the immunosuppressive domain (ISD). HERV3.1 was also upregulated in cisplatin resistant cells. Although this HERV has lost its fusogenic properties, the immunosuppressive activity has been retained because the ISD is highly similar to the CKS-17 immunosuppressive peptide.

### The neuronal morphology of SKOV3 cells exposed to cisplatin correlates with the expression of neuronal markers

Gene expression analysis using qPCR and NGS of SKOV3^WT^ and SKOV3 cells exposed to cisplatin for 72 h revealed a clear switch from an epithelial to a mesenchymal and from this to an ectodermal state. Nevertheless, these findings suggest that the conversion of mesoderm-derived cell type like ovarian carcinoma cells into a neuronal type had occurred at the transcriptional level.

**Fig. 5:**
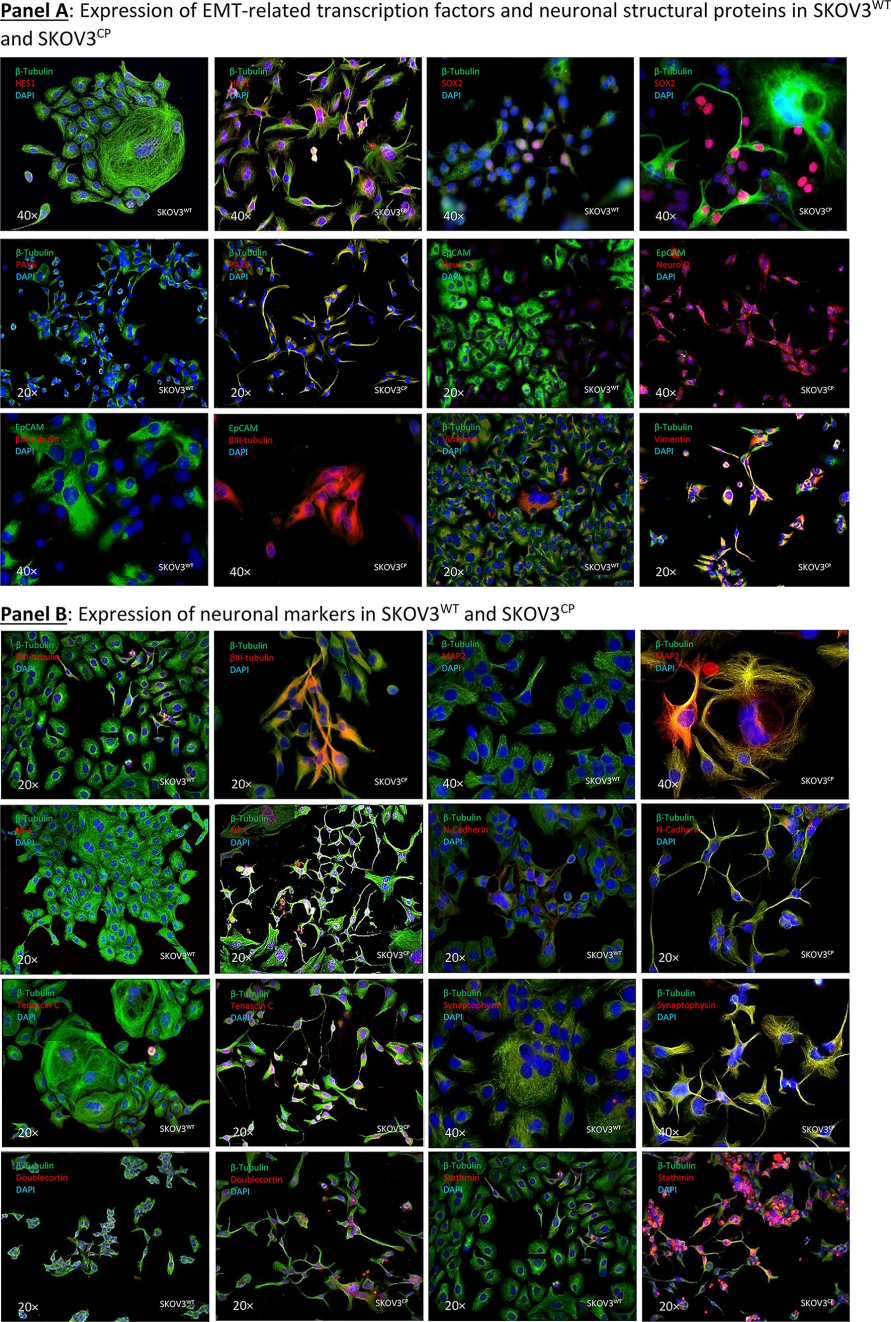

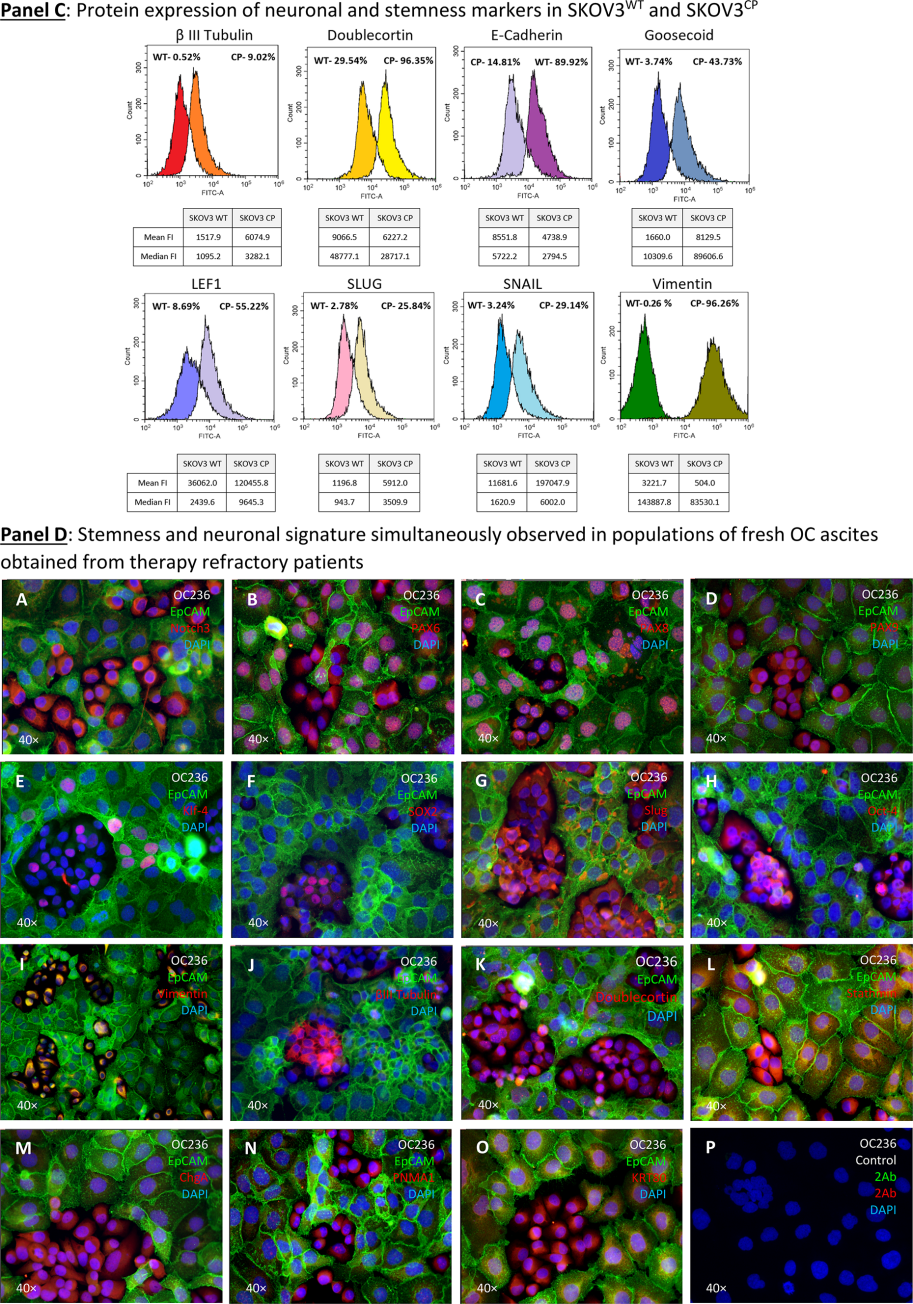

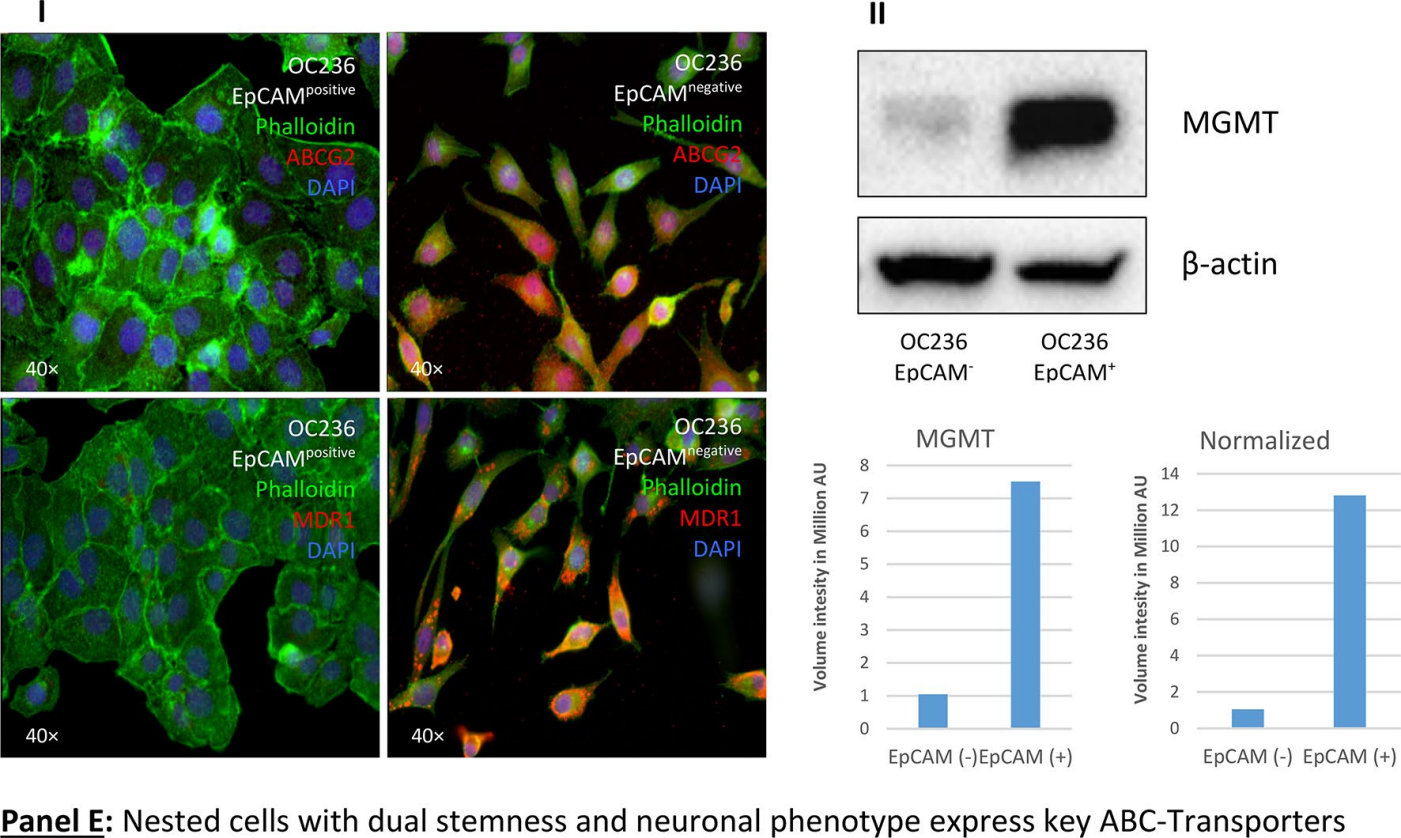
Expression of EMT-related transcription factors, neuronal structural proteins and MDR in SKOV3^WT^ and SKOV3^CP^. **Panel A**: expression of cardinal transcription factors defining a neuronal phenotype and structural proteins. The basic helix-loop-helix (bHLH) HES1, the SRY (sex determining region Y) box 2 also known as SOX2, the paired box protein PAX6 and the basic helix-loop-helix transcription factor NeuroD, all well-known key players in neuronal development, are upregulated in SKOV3^CP^ cells. Some of them like HES1, SOX2, and PAX6 show transcriptional upregulation in qPCR analysis. By the same token, structural proteins βIII-tubulin and Vimentin are upregulated, whereas the epithelial marker EpCAM is downregulated. **Panel B**: overexpression of important neuronal markers like MAP2, NF-L, N-cadherin, Tenascin C, Synaptophysin, Doublecortin and Stathmin in SKOV3^CP^. Importantly, the expression of these markers correlates with the neuronal morphology of the cells. **Panel C**: flow cytometric expression analysis of the EMT-related markers E-cadherin, Vimentin, Snail and Slug and the neuronal markers Doublecortin and Goosecoid. **Panel D**: stemness and neuronal signature in primary OC cells cultivated in 2D. Clusters of EpCAM negative cells with concurrent stemness and neuronal signature are observed. These nested cells are positive for different transcription factors (TFs, **A**-**H**) which are considered strong contributors to stem cell maintenance and cell differentiation. Of note, most TFs are mainly found in the cytoplasm of cells instead in the nucleoplasm, with the exception of PAX8, which is also localized to the nucleus (for reference, the RGB decomposition of pictures attached in the Supplemental Information). It is evident that those nested cells are not of epithelial nature by the lack of EpCAM and the expression of Vimentin (**I**), a structural protein typical for mesenchymal cells. Notably, those cells significantly express several neuronal markers like βIII-tubulin (**J**), a neuron-restricted microtubule protein, Doublecortin (**K**), Stathmin (**L**), the neuroendocrine marker Chromogranin A (ChgA) (**M**) and the dually neuron- and testis-specific protein PNMA1 (**N**). Interestingly, the type II intermediate filament Keratin 80 (KRT80) (**O**) was expressed in these nested cells, indicating that this filament is involved in early stages of tumor cell differentiation. **P**, secondary antibody control. **Panel E**: multidrug resistance in cells with stemness and neuronal phenotype. Nested cells isolated from primary cultures from ovarian carcinoma ascites express a multidrug resistance phenotype as judged by the expression of MDR1 and ABCG2. Cells were sorted into EpCAM positive and negative populations and analyzed by immunofluorescence. **II**: Expression of MGMT (Methylated-DNA–protein-cysteine methyltransferase) DNA repair enzyme in OC236, sorted by EpCAM expression. The EpCAM negative cells, which have a stemness/neuronal phenotype revealed no expression of MGMT whereas the EpCAM positive cells (epithelial) exposed an upregulation of this enzyme. This indicates that the EpCAM positive cells are more resistant to agents like cisplatin. ICC results from n = 6 experiments, FACS results from n = 3 experiments. Complete FACS histograms data can be found in Supplemental, Fig. 1.

To determine if effector proteins are synthesized by cells challenged with cisplatin, we investigated the transcript-protein correlation and observed that the protein expression of relevant neuronal markers was, in fact, congruent with the gene expression data (Fig. 5, Panels A, B and C).

Figure 5, Panel A reflects the expression of key transcription factors crucial for neuronal development, e.g. SOX2 and HES1 TFs, both involved in the generation and maintenance of progenitor and stem cells. Notably, HES1 is vital for nervous system development, influencing neural stem cells and neuroepithelial cells by repressing basic helix-loop-helix (bHLH) activators^42,43^. The transcription factor PAX6, which regulates neurogenetic and neural stem cell self-renewal processes, was overexpressed^44^. The bHLH transcription factor NeuroD, which is a critical controller of the neuronal vs. glial fate decision and responsible for dendrite formation and maintenance, was overexpressed in SKOV3^CP^ cells^45,46^. The occurrence of EMT in the SKOV3^CP^ cells can also be inferred by the loss of the epithelial marker EpCAM and the gain of Vimentin, a type III intermediate filament (IF) protein exclusively expressed in mesenchymal cells.

Ovarian carcinoma cells exposed to cisplatin also overexpressed certain structural proteins which are characteristic of neuronal tissues. Figure 5, Panel B reflects the increase in several cytoskeletal structural proteins like βIII-tubulin and MAP2 in SKOV3^CP^ cells. Both are almost exclusively expressed by neurons and play important physiological roles in neuron development^47,48^. In addition, the low molecular weight neurofilament protein (NF-L) is clearly upregulated in SKOV3^CP^ cells. This neurofilament is mainly localized in the axonal regions of neurons where it helps to stabilize the axons and facilitates axonal transport^49,50^. N-Cadherin, which plays a decisive role during neural development^51^, was also overexpressed in ovarian cancer cells treated with cisplatin. However, the expression of N-Cadherin mediates the migration of mesenchymal cells from the bone marrow towards tumors, which is associated with poor prognosis^52,53^. Stathmin, also known as oncoprotein 18, plays a crucial role in cytoskeleton regulation specifically by modifying microtubule dynamics. This protein was overexpressed in SKOV3^CP^ cells. The expression of Stathmin is upregulated during neuronal development^54,55^ and has been associated with carcinogenesis and metastatic processes^56,57^. Furthermore, Tenascin C, which is expressed in the extracellular matrix in neurogenic domains of the central nervous system and also temporarily during organogenesis, was overexpressed in SKOV3^CP^ cells^58,59^. Tenascin C expression is clinically associated with the metastatic process^60,61^. Some neuronal transmitters use Synaptophysin as a mediator. This synapse-associated protein was overexpressed in SKOV3^CP^ cells. Importantly, Synaptophysin is expressed in neuroendocrine cells or neurons^62,63^.

Using flow cytometry (Fig. 5, Panel C, and Figure SF9), we investigated the expression or loss of certain EMT markers in SKOV3^CP^ cells vs SKOV3^WT^. The expression of proteins indicative of EMT was correlated to a significant reduction of E-Cadherin and increase of Vimentin (to approx. 75% and 96%, respectively, Fig. 4, Panel C). Likewise, the EMT-initiating factors Snail and Slug were upregulated in about 29% and 23% respectively. A notable finding was the upregulation of Doublecortin, a key protein expressed by neuronal precursor cells. Moreover, together with the upregulation of Goosecoid, a protein involved in driving EMT, chemotherapy refraction and metastasis formation was observed^64^.

### Cells isolated from OC ascites revealed potential origins of the tumor

It is necessary to consider that the majority of commercial OC cell lines were derived from cisplatin refractory patients and therefore not truly virgin to this therapy. Even though cell lines provide valuable research models, they do not recapitulate the tumor biology as primary cell cultures do. For this reason, we sought to investigate the existence of cells with a neuronal signature in fresh cells isolated from ascites from ovarian carcinoma patients.

Figure 5, Panel C (merged channels) and Figure SF10 (RBG decomposition) shows the growth features and the expression of different markers in non-treated OC cells isolated from ascites. The first characteristic observed in this primary cell culture is the existence of islands of nested cells, which depict a distinct morphology from the surrounding cells.

Nested cells express a vast number of pivotal transcription factors typically observed in embryonic development. Notch 3 is mainly expressed in the nested cells (Fig. 5, Panel D, A). PAX6 and PAX8 were expressed in both nested and their adjacent cells (B & C). PAX9 was highly expressed by nested cells and to a lesser extent in surrounding cells. Only few cells express the TFs Klf-4 and SOX2 (E & F), mainly detectable in the nested cells. Contrarily, Slug and Oct-4 are strongly expressed in nested cells (G & H) with Oct-4 being restricted to these cells, while Slug is also detectable in adjacent cells.

It is evident that nested cells show an EMT signature, indicated by the exclusive localization of Vimentin in these cells (I). Their neuronal nature is further revealed by the expression of βIII-tubulin (J), Doublecortin (K), Stathmin (also found in the mixed populations) (L), the neuroendocrine marker ChgA (M) and PNMA1 (N). Intriguingly, nested cells also overexpress keratin 80 (KRT80) (O), an epithelial related keratin. However, KRT80, despite being an epithelial keratin, is clearly expressed in the nested cells, indicating that this keratin may also serve as a marker for cells in an EMT transit. However, this was previously observed in the context of colorectal carcinomas and associated with high metastatic potential^65^. Consequently, it raises the question of whether this keratin is also shared by cells with a neuronal signature.

In summary, these findings suggest two key points i: the nested cells in OCs exhibit a strong neuronal signature, indicating that the tumors surely arose from this cell type and ii: these cells are in fact the cancer stem cells based on the expression of plethora of TFs which are cardinal for both stem cell maintenance and cell differentiation.

### The nuclei of cells undergoing an EMT are impermeable to pivotal transcription factors

In OC236 cell model, PAX8 TF is observed without restriction in both nucleus and cytoplasm, contrary to PAX6, PAX9 and Notch3 (Fig. 5, Panel C, merged channels and Figure SF10, RBG decomposition). This cytoplasmic restriction was also observed for PAX6 in cisplatin resistant SKOV3^CP^ cells (Fig. 5, Panel A). Consequently, this TF is the only one that can properly regulate the transcription of its downstream target genes. PAX8 is involved in the embryonic development of the thyroid gland, specifically its rudimentary lateral portion, which develops from neural crest cells.

Concerning the subcellular localization of PAX TFs in particular, it is true that in “neuronal” or stem cells, such TFs are ectopically found in the cytoplasm instead in the nucleoplasm as expected. We verified that both gene expression and protein synthesis of these TFs occurred and that at the protein level they remain functional. In this regard, the more epithelial the cells appear, the more likely it is to find PAX TFs in the nucleoplasm. Conversely, in cells depicting a “neuronal” or adult stem cell phenotype, PAX TFs are less likely to be located in the nucleoplasm (Fig. 5, Panel C, merged channels and Figure SF10, RBG decomposition). This clearly indicates that the nuclear membranes of both populations are functionally different, with those nuclei from cells that are phenotypically more stem or “neuronal” being less permissive to these TFs, fact that was unknown until now. This is an important observation that merits further biochemical studies in order to elucidate the underlying mechanism. If these cells do not utilize these TFs themselves, it is possible that they export them to other target cells.

In summary, we have established that several proteins, which are biologically characteristic of neuronal development, were overexpressed in ovarian carcinoma cells treated with platinum-derived drugs. Importantly, the cell shape was consistent with a neuron-like morphology, indicating that the upregulation of the neuronal proteins was partly responsible for the observed changes in cell shape. Moreover, as already shown in Fig. 4, the gene expression of neuronal markers strongly correlated with the protein synthesis. We have thus demonstrated a relationship between protein expression and the acquisition of a neuronal-like morphology in ovarian carcinoma cells treated with platinum compounds.

### Are “twin” cells the source of the conversion of epithelial to neuronal-like cells upon platinum- derived drugs exposure?

We hypothesized that a naturally recurrent cell type, described above and called “twin”, present among SKOV3^WT^, OVCAR-3 cells and primary ovarian carcinomas obtained from ascites (Figure SF5 & SF6), might be the source of the expansion of cells with neuron-forming capacity (Fig. 3, Panel A). These cells maintain the dual formation for long periods; especially in cisplatin-exposed cultures (see Videography C & D).

**Fig. 6:**
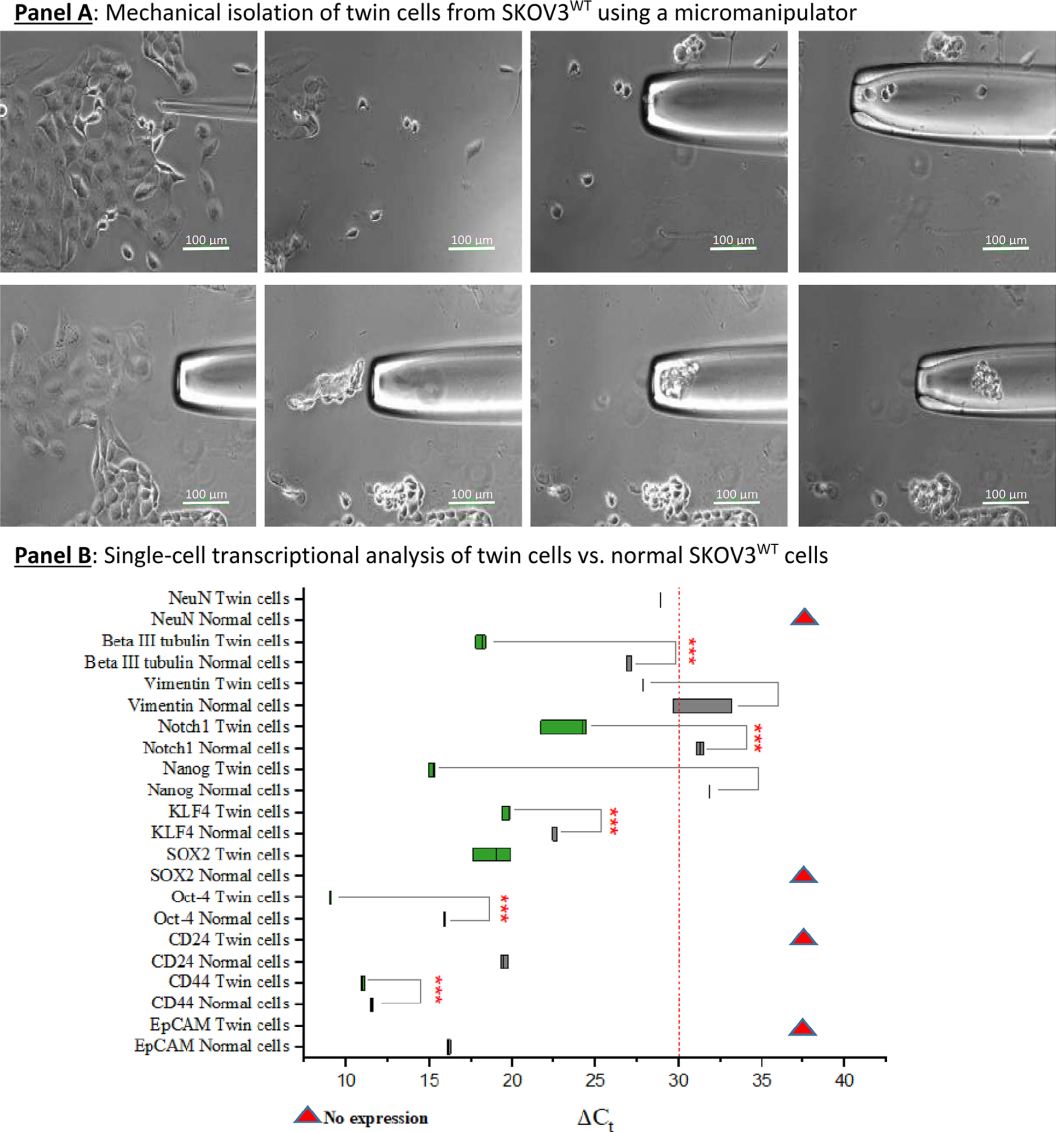

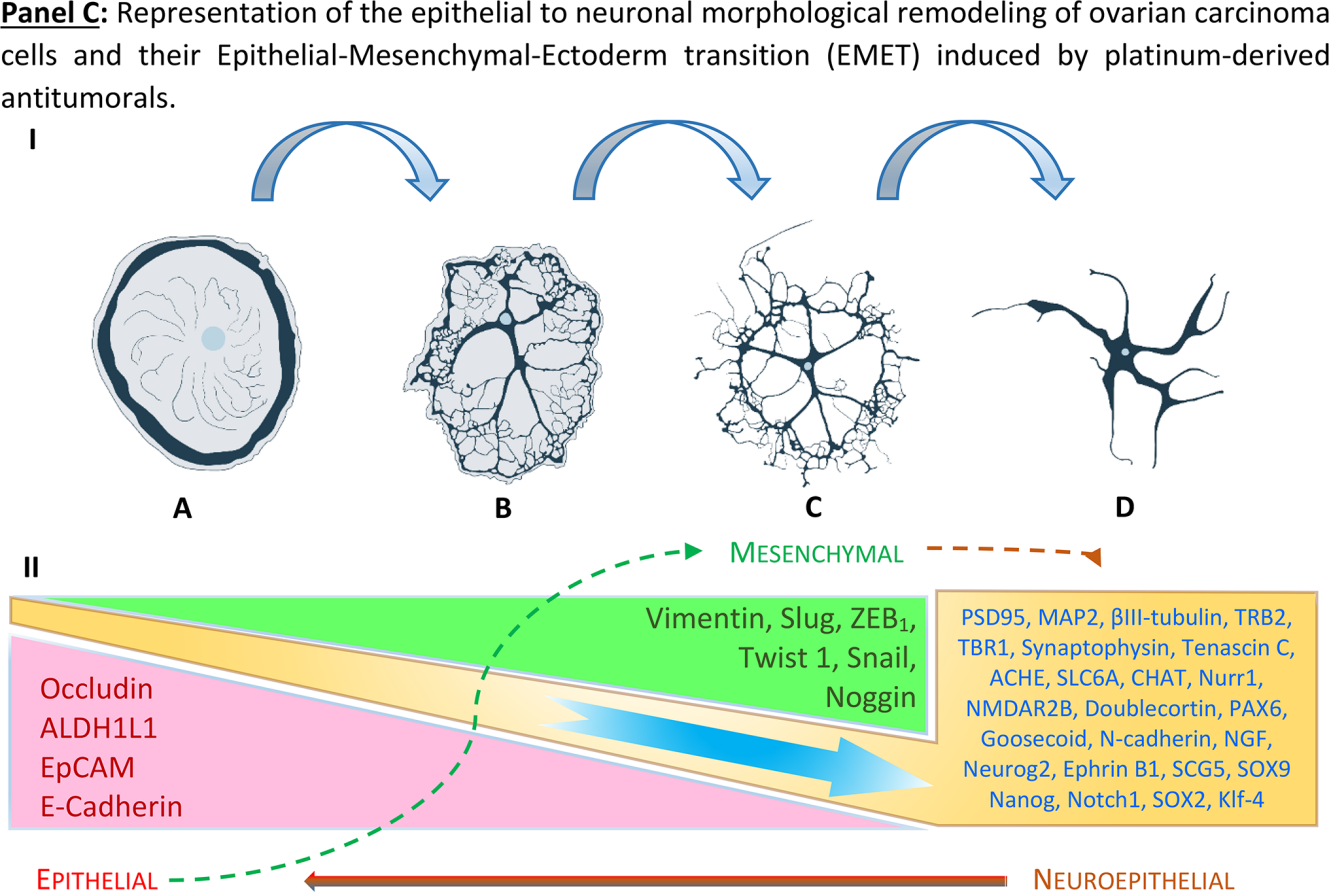
Isolation and analysis of “twin” cells present in SKOV3 cultures. **Panel A**: “twin” cells as shown in Fig. 3 (**Panel A**, **A**-**D** and **Panel B**, **A**-**C**) were subjected to mechanical isolation using a one-arm micromanipulator. At least 20 “twin” cells and a proportional amount of surrounding normal cells were collected for single-cell RNA isolation. **Panel B**: qPCR transcriptional analysis of “twin” cells vs. normal epithelia-shaped cells. A dual neuronal-stem signature is detected at the transcriptional level. Some markers were not detected (red triangles): NeuroN, SOX2 in normal cells and CD24 and EpCAM in “twin” cells. **Panel C**: **I**: Cartoon representing OC cells acquiring a neuron-like morphology under the influence of platinum-derived compounds as seen in Fig. 1. A large cell (**A**) gradually condenses its cytoplasm and starts forming dendritic tree-like structures (**B** & **C**). The cytoplasm is finally dismantled until fully formed dendrite-like structures are left (**C** & **D**). These morphological changes correlate with the expression of a plethora of neuronal-defining markers. **II**: Diagrammatic representation of the modulation of pathways involved in EMT and from this point to a “neuro-mesenchymal” inter-condition or EMET based on the transcriptional and protein expression profiles. SKOV3 cells have a basal expression of several neuronal markers, which is enhanced by exposure to sub-toxic concentrations of platinum-derived drugs. The expression of cardinal stemness markers like Nanog, Notch1, SOX2, and Klf-4 suggests the acquisition of cancer stem cells status. The cells that have moved to a neuroepithelial condition are in a transitory status of dormancy. Results are representative of n = 3 experiments.

To explore this hypothesis, we isolated “twin” cells using a micromanipulator and analyzed their stemness and neuronal gene expression at the single-cell level (Fig. 6, Panel A). We collected about 20 cells during cell division, extracted their RNA, and performed qPCR transcriptional analysis. A limit of 29 cycles was established, with ΔCt values above this limit excluded from consideration. However, we reported genes that showed significant expression in at least one cell type, either SKOV3^WT^ or “twin” cells. This was the case with NeuroN and SOX2, which were not detectable in wildtype cells but were detected in “twin” cells. In line with this, both CD24 and EpCAM were not detected in “twin” cells, but were highly expressed in SKOV3^WT^ cells (Fig. 6, Panel B). The (CD44^+^/CD24^-^) SKOV3-derived “twin” cells lack expression of CD24, often associated with cancer stemness. It is worth mentioning that CD24, though specific to B-lymphocytes, remains ambiguous as a definitive stemness marker due to tumor heterogeneity and its inconsistent expression across tumor types^66^.

Single-cell RNA analysis further confirmed that “twin” cells possess stemness characteristics alongside the expression of neuronal genes (Fig. 6, Panel B). In addition, essential transcription factors involved in self-renewal, pluripotency and/or neurogenesis were significantly upregulated, including Klf-4, Oct-4, SOX2, Nanog and Notch1 being the most prominent ones. Moreover, the transition from an epithelial to a neuronal-like steady state was confirmed by the loss of EpCAM (epithelial), the gain of βIII-tubulin (neuronal) and Vimentin (see Figures SF10-13). The expression of such genes was confirmed in ovarian carcinoma primary cultures (Figure SF14).

“Twin” cells likely represent a cell type with dual characteristics: both stem and neuronal. Nevertheless, while the entire population of SKOV3 and other ovarian carcinoma cell lines exhibit a neuronal background at the protein and transcriptional levels (summarized in Fig. 6, Panel C), exposure to platinum compounds unmask this signature. However, this “neuronal” background and the more defined neuron-like signature caused by cisplatin exposure probably do not render in functional neurons at this stage, since preliminary experiments for the excitatory physiology of mature neurons were unsuccessful (data not shown). Thus, the shift to a neuronal condition is incomplete.

### Investigating the neuroepithelial origin of ovarian cancers in ovarian tissues.

The precise anatomical origin of serous ovarian carcinoma remains a topic of considerable debate. While some evidence suggests that OCs may arise from the terminal portion of the fallopian tube or the ovarian surface epithelium, a definitive explanation of the specific cell type undergoing malignant transformation has been elusive^67,68^.

**Fig. 7:**
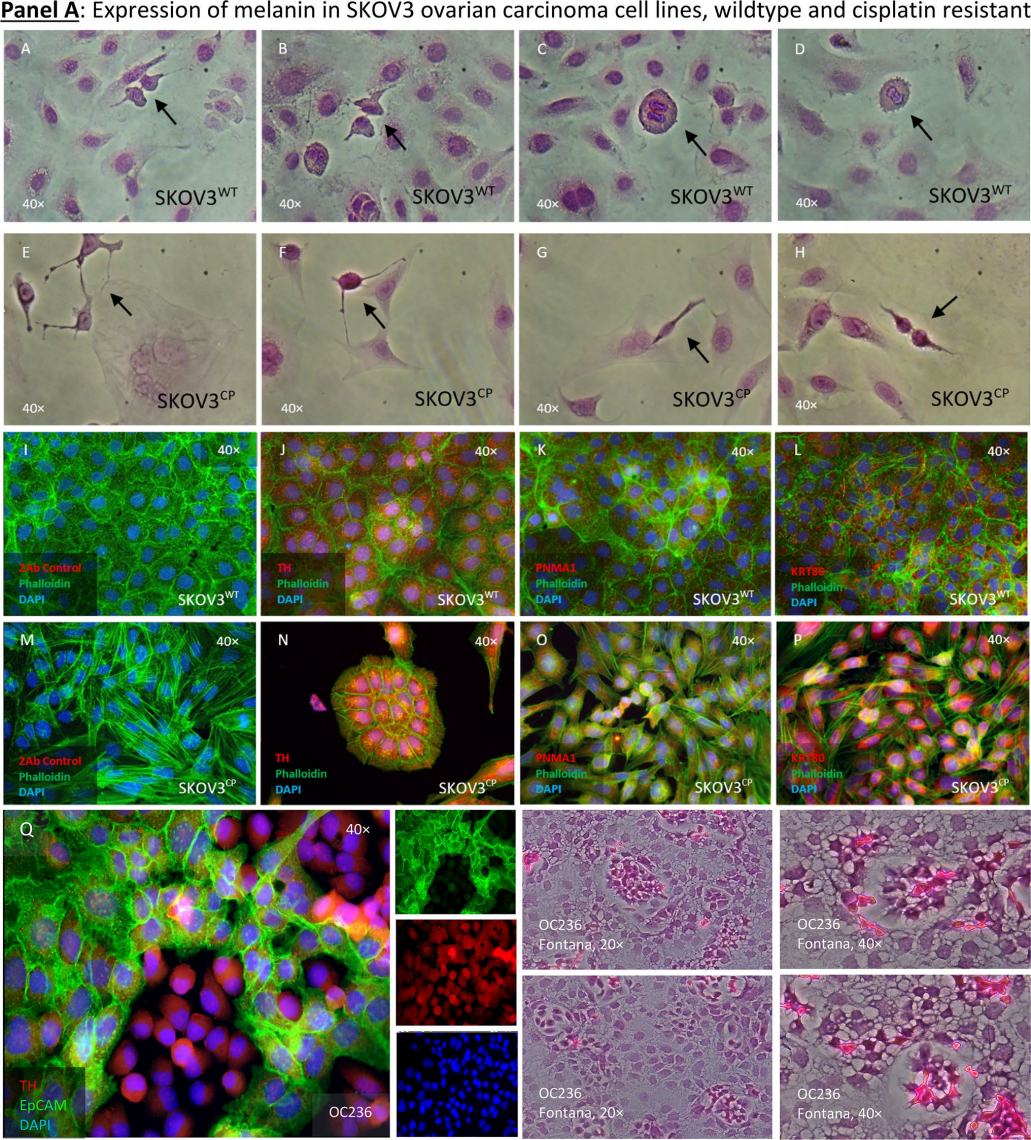

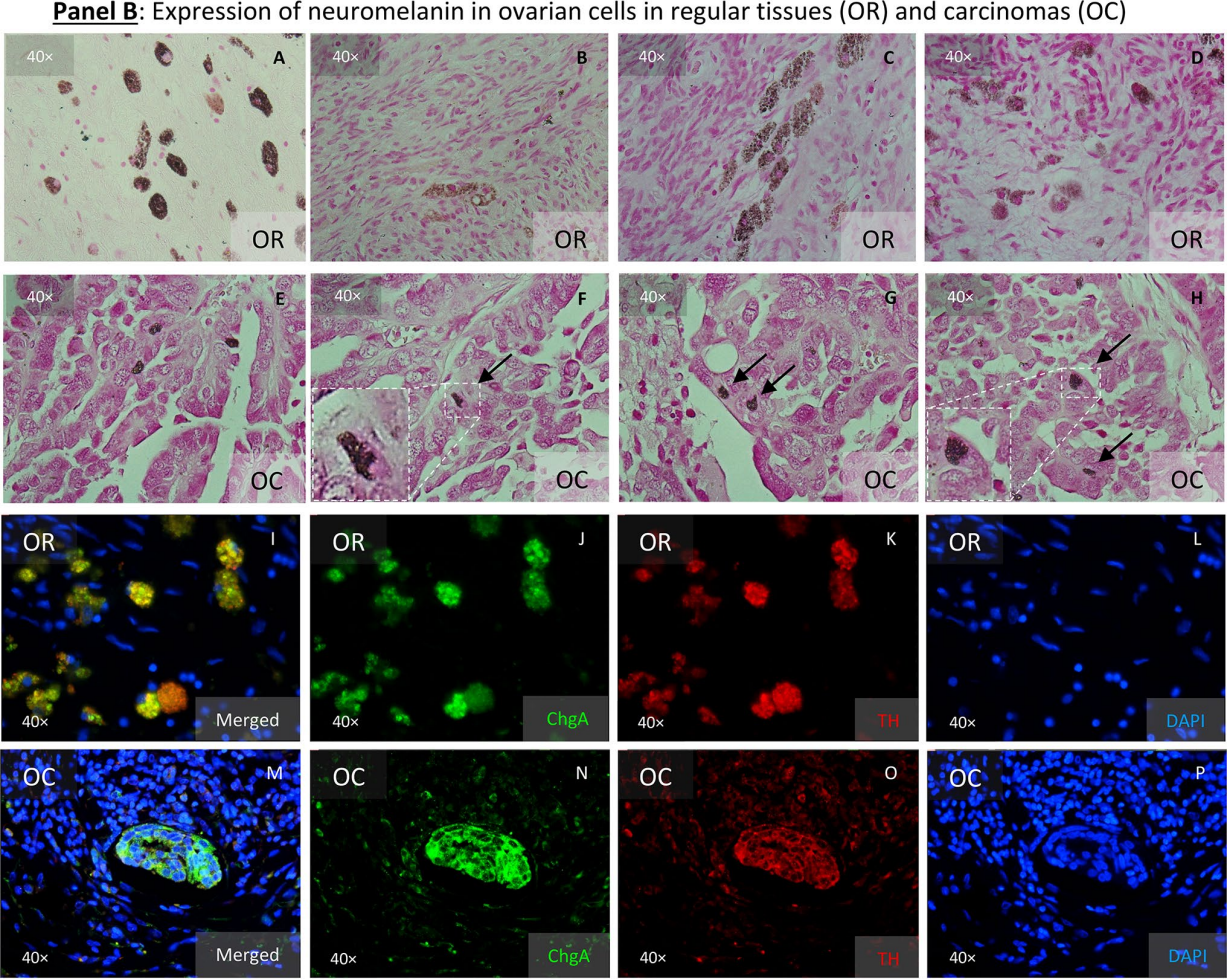

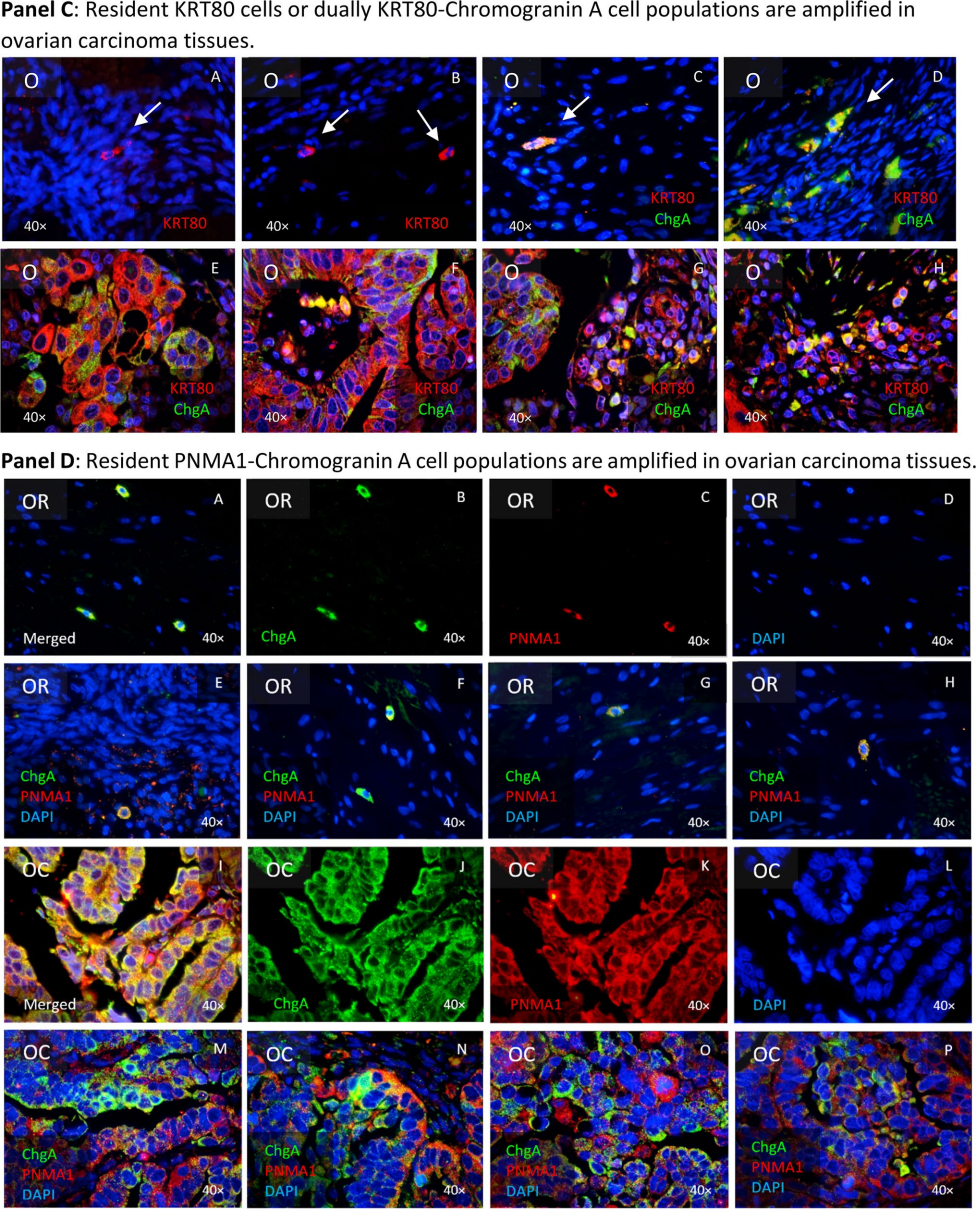
Expression of neuronal-related markers by cells from ovarian regular as well as from tumoral tissues. **Panel A**: Neuromelanin-expressing cells in SKOV3 wildtype and its cisplatin resistant subline. Fontana-Masson staining reveals the presence of black/brown pigmented granules, consistent with melanin producing cells both cell types (**A**-**H**). “Twin” cells appear to express more pigmentations that the surrounding cells (black arrows). The staining with anti-Tyrosine hydroxylase (TH) confirmed that the argentaffin staining reveals the expression of neuromelanin (**J** & **N**) as detected using the Fontana-Masson staining. Cisplatin resistance appears to enhance the expression of this enzyme as noticeable by ICC staining (ICC: Phalloidin in green, TH in red and DAPI in blue). The paraneoplastic protein MA1 (PNMA1) (**K** & **O**) is also detectable in SKOV3 ovarian carcinoma cells. Keratin 80 (KRT80) (**L** &**P**) appears enhanced in cells exposed to cisplatin for 72 h (**P**). Secondary antibody control (**I** & **M**). Expression of TH in an OC primary cell culture (**Q**) and the argyrophilic cells in these cells as detected by Fontana-Mason staining (**R**). **Panel B**: Argentaffin cells in regular (**A**-**D**) and tumoral ovarian tissues (**E**–**H**), as revealed employing the Fontana-Masson staining. In ovarian carcinoma tissues, argentaffin cells looks smaller than in the normal tissues. Few cells dually positive for TH and Chromogranin A (ChgA) are detected in regular ovarian tissues (**I**-**L**), whereas massive amplification of both antigens are detected in ovarian carcinoma tissues (**M**-**P**), as detected by IHC techniques (ICC: ChgA in green, TH in red and DAPI in blue). **Panel C**: It is possible to discriminate in regular ovarian tissues two different cell populations: one cell type lonely express KRT80, whereas the other cell type dually express KRT80 and ChgA (**A**-**D**). It appears however, that those cell types are extremely amplified in tumoral ovarian tissues, as revealed by IHC (**E**–**H**). **Panel D**: In regular ovarian tissues, some few ChgA-PNMA1 resident cells are noticeable (**A**-**H**). The expansion of the same cell phenotype is predominant in tumor areas (**I**-**P**), constituting the majority of cells. Paraffin sections representative for n = 10 patients. Magnification 40 × .

Given the extensive data showing that ovarian cells can undergo morphological and gene expression changes towards an ectodermal state under cisplatin exposure, we aimed to identify cells with distinct neuronal-related features in ovarian tissues. We suspected that the darkness of some cells among 3D cell cultures (hanging drops, data not shown) could be a result of melanin pigments, therefore we employed the Fontana-Masson silver staining commonly used in histopathology to detect argentaffin pigments, including melanin. This staining revealed that nascent “twin” cells were particularly positive for argentaffin (Fig. 7, Panel A, pictures A-H). However, since this method does not distinguish between types of melanin, we looked for the expression of Tyrosine hydroxylase (TH), a key enzyme in the production of neuromelanin (NM) and catecholamines in the central nervous system (CNS), peripheral sympathetic neurons and the adrenal medulla Our findings indicated that TH expression was limited to a few cells in SKOV3^WT^ cultures but was significantly increased in cells exposed to cisplatin (Fig. 7, Panel A, pictures J vs. N). Likewise, the PNMA1 neuronal marker was also overexpressed in cisplatin treated cells (Fig. 7, Panel A, pictures L vs. P). In addition, in the primary OC cells OC236, TH was primarily detected mainly in nested cells (Q) which also exhibited stronger Fontana-Masson staining (R).

We then scrutinized ovarian tissues, both regular (non-tumoral) and tumoral for argentaffin cells or regions using the above mentioned histochemical method. We detected argentaffin cells in the ovarian surface epithelium of regular tissues (Fig. 7, Panel B, upper block of histochemical pictures, OR -ovarian regular-, A-D). These cells appeared morphologically larger compared to the surrounding cells and presented stronger pigments. In contrast, in tumoral tissues, argentaffin cells were not as concurrent and the few ones that were found appeared smaller and more dispersed (Fig. 7, Panel B, upper block of histochemical pictures, OC –ovarian carcinoma-, E–H). Notably, the distribution of TH expression mirrored that of Fontana-Masson staining in normal tissues (Fig. 7, Panel B, inferior block of IHC pictures, OR, I-L). However, TH was also specifically expressed in tumor areas, forming distinct islands (Fig. 7, Panel B, inferior block of IHC pictures, OC, M-P) matching the expression of the neuroendocrine marker ChgA. This discrepancy between the staining patterns led us to hypothesize that melanin pigments might be subject to tissue clearance via degradation, despite the noticeable expression of TH.

These observations reinforced the neuroepithelial nature of OCs, but the precise origin of “neuronal-like” cells contributing to tumor proliferation remained unclear. The experiments with Fontana-Masson staining and TH expression alone were insufficient to conclusively classify these cells as neuronal. Further investigation revealed that some rare cells in normal ovarian tissues expressed KRT80, a keratin previously associated with epithelial tissues, alongside ChgA, suggesting a potential neuroendocrine function (Fig. 7, Panel C, upper block of IHC pictures, OR A-D). It is worthy to mention, that ChgA is an activatable protein, functioning as an on/off switch in response to specific stimuli, rather than constitutively produced. We found a significant amplification of KRT80-ChgA dual-positive cells, indicating that these rare cells in non-tumoral ovarian tissues could be the progenitors that expand to form the tumor mass (Fig. 7, Panel C, upper block of IHC pictures, OR E–H). Moreover, we found that PNMA1-ChgA double positive cells were significantly amplified in the ovarian carcinoma tissues (Fig. 7, Panel D, I-P). However, in regular ovarian tissues, these cells are rarely found (Fig. 7, Panel D, A-H).

### Notch inhibitors are insufficient to prevent “axonal or dendritic arborization” in SKOV3^CP^ cells

Notch receptors are key regulators of signaling pathways that influence various biological processes, including embryogenesis, maintenance of neuronal progenitor cells and neurite development. Notch signaling also plays a role in the cellular plasticity necessary for the upkeep of the nervous system and ongoing neurogenesis in adult tissues. To investigate the possible interference of Notch signaling with the observed “axonal or dendritic arborization” in induced neuronal-like OC cells, we treated SKOV3^CP^ cells with two GSIs, both indirect inhibitors of this cascade [RG-4733 (1 µM) or DAPT (5 µM)] together with 2 µg/mL of cisplatin for 5 days. We previously corroborated that these compounds inhibit Notch pathway, since HES1 was downregulated under the influence of both small molecules (Figure SF15A). Despite the inhibition of Notch signaling, there were no significant changes in the morphology of the treated cells; they retained the same “neuronal” morphology observed in untreated cells (Figure SF15B). This indicates that Notch signaling does not play a role in driving the dendritic “arborization” process in this cell model. These findings are consistent with previous observations that Notch does not translocate to the nucleus in the nested cells of the OC236 primary cells.

Curiously, in Neuropan medium (Fig. 3, Panel B), which is commonly used to develop neuronal tissues, the Notch1 appears downregulated and the cells gained the “neuronal” morphology. Consequently, it appears that this “arborization” in OC is independent from Notch signaling.

### Stemness potential of nested cell population in OC236

Nested cells represent a unique cellular subpopulation capable of binary differentiation: on one hand, they maintain the condition of cancer stem cell in a small fraction (probably via multinucleation) as seen by the expression of stem cell markers like Oct-4, SOX2 and Klf-4 and, on the other hand, they differentiate towards an epithelial condition as seen by gradual loss of mesenchymal characteristics in parallel with the acquisition of the epithelial phenotype, thus shaping the cellular heterogeneity present in tumors (Fig. 5, Panel D, SF10). It should be noted that over time, the population of nested cells gradually decreases, with these cells becoming dispersed among the predominant epithelial cells. Though this could be the results of different grow kinetics.

### Interfering Notch and MAP4K4 signaling pathways render in more neuronal background in OC cells

To confirm the activity of both RG-4733 and DAPT in wildtype SKOV3 and OVCAR-3 cells, we monitored the expression of HES1, whose expression is governed by the Notch pathway. Indeed, both inhibitors repressed HES1 expression (**see SF15A**).

**Fig. 8:**
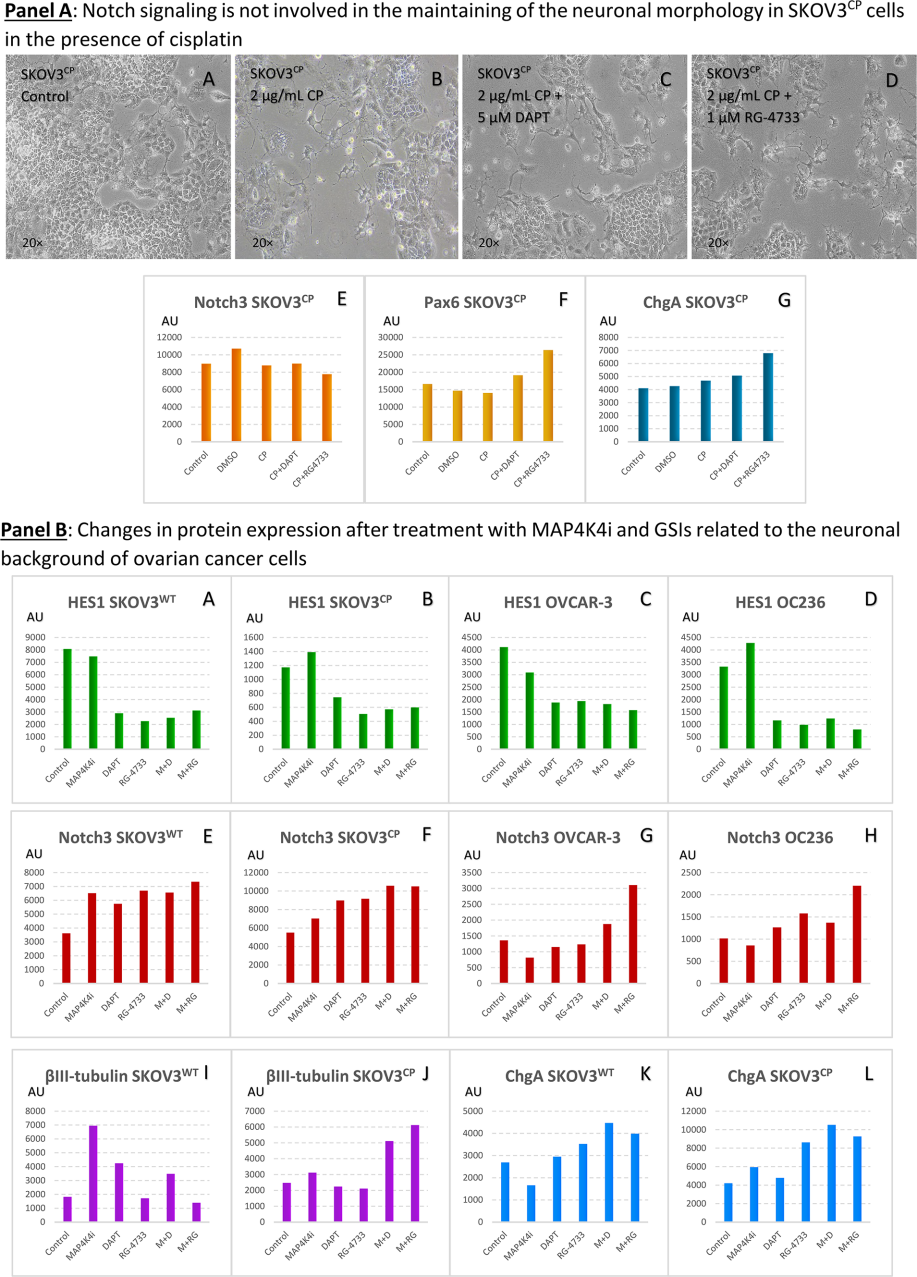
Interfering Notch and MAP4K4 signaling pathways in OC cells. Panel A: combination treatment of cisplatin and GSIs. SKOV3^CP^ cells were treated under the following conditions: (**A**) medium control, (**B**) 2 µg/ml cisplatin, (**C**) 2 µg/ml cisplatin + 5 µM DAPT, and (**D**) 2 µg/ml cisplatin + 1 µM RG-4733 for 120 h. The simultaneous administration of cisplatin and GSIs did not reverse the neuronal morphology of SKOV3^CP^ cells (**A**-**D**). Additionally, this combination did not lead to significant changes in Notch3 expression (**E**). However, the incubation with both GSIs induced an upregulation of PAX6 and ChgA, with RG-4733 exerting the most pronounced effect. **Panel B**: combination treatment of MAP4K4i and GSIs in OC Cells. The exposure of OC cells to MAP4K4i did not induce general changes as expected in HES1 protein levels across various OC cell lines. However, both GSIs significantly downregulated HES1 protein expression (**A**-**D**). Notch3 expression was stimulated by all inhibitors in both SKOV3^WT^ and SKOV3^CP^ cells (**E**). In OVCAR-3 and primary OC cells OC236, only the combination of MAP4K4i with RG-4733 synergistically enhanced Notch3 expression (**G**-**H**). Furthermore, βIII-tubulin expression was strongly stimulated by the MAP4K4i in SKOV3^WT^ cells, an effect not observed in the resistant SKOV3^CP^ subline. Nevertheless, the combination of MAP4K4i with GSIs led to an enhanced expression of this neuronal-related structural protein (**I**-**J**). ChgA expression was slightly repressed in SKOV3^WT^ cells but enhanced in SKOV3^CP^ cells after 24 h of MAP4K4 inhibitor treatment. Both GSIs increased ChgA expression, and this effect was slightly potentiated in the presence of the MAP4K4 inhibitor (**K**-**L**). **Abbreviations: M**: MAP4K4i; **D**: DAPT; **RG**: RG-4733; **AU**: arbitrary units. Bar graphs represent densitometric analyses of western blots performed to measure protein expression following drug exposure. Results are representative of n = 3 independent experiments.

In SKOV3^CP^ cells, the simultaneous incubation of cisplatin and GSI do not revert their neuronal morphology (Fig. 8, Panel A, A-D and SF15B). The expression of Notch3 remains unaltered regardless the type of treatment (E). Both small molecules RG-4733 and DAPT effectively stimulate the protein levels of both PAX6 and ChgA (F & G). Consequently, both GSI enhance the neuronal background in the resistant subline SKOV3^CP^.

We next sought to study the effect of MAP4K4i and GSI on OC cells in the absence of cisplatin. The MAP4K4i revealed ambiguous expression of HES1 in different OC cells. Nevertheless, both GSI alone or in combination with the MAP4K4i effectively repressed the expression of HES1 (Fig. 8, Panel B, A-D). In SKOV3 cells, wildtype and cisplatin resistant all drugs alone or in combination stimulated the expression of Notch3 at protein level in absence of cisplatin (Fig. 8, Panel B, E–F). Contrarily, a synergistic or additive effect of the MAP4K4i and both RG-4733 and DAPT, GSIs on Notch3 expression was detected in OVCAR-3 and the primary ovarian carcinoma cells OC236 (Fig. 8, Panel B, G-H).

Interestingly, the inhibition of MAP4K4 and Notch pathways induces the upregulation of both βIII-tubulin and ChgA, two classical neuronal markers. This was observed in SKOV3^WT^ and more accentuated in the cisplatin resistant SKOV3^CP^ subline (Fig. 8, Panel B, I-L).

## Discussion

Platinum-based drugs (cisplatin, carboplatin, and oxaliplatin) are metal complexes commonly used as a cornerstone in chemotherapy regimens designed to treat various malignancies, including ovarian cancer. However, their potent antitumor properties come with significant side effects, including nephrotoxicity, neurotoxicity, and hematopoietic toxicity^69^. These therapeutics also drive cancer cell heterogeneity and the emergence of chemorefractory clones with metastatic properties acquired e.g. via EMT by upregulation of transcription factors like HIF1α, Snail, Slug or Twist 2^70,71^. However, little is known about the capacity of platinum-based cytostatics to convert ovarian cancer cells into neuron-like cells and, importantly, their molecular characterization, as well as the physiological fate of this induced neuronal cell type. Other groups have reported the conversion of ovarian carcinoma cells into different cell types via endomitosis/multinucleation after exposure to CoCl_2_ (a hypoxia-mimicking compound) and cisplatin^25^. This effect implies a retrograde differentiation back to the original cells from which the three embryological layers have diverged. Our primary interest was therefore to evaluate the acquired neuronal status of ovarian carcinoma cells after exposure to sub-toxic concentrations of cisplatin.

The fallopian and fimbria tubes are naturally constructed with circular and longitudinal smooth muscles, and where muscles exist, associated neuronal structures are typically present as well. In fact, our results obtained from the transcriptome analysis revealed that the expression of some genes related to the neuromuscular junction are expressed in SKOV3 wildtype ovarian carcinoma cells and overexpressed in SKOV3 cells exposed to cisplatin^72–74^. Interestingly, within the regular ovarian surface epithelium (OSE), we identified cells exhibiting neuronal features, which are prominently observed in ovarian carcinoma cells, as reported in this study. The related question is whether the cells of origin in ovarian carcinoma are a kind of neural (or neuroepithelial) cell type residing in the fimbria, at least for the serous type, given that the SKOV3 or OVCAR-3 cell models employed in this work are derived from an ovarian serous cystadenocarcinoma. The work of Giuseppe Testa’s lab provide important clues on this possibility^68^, since their studies revealed that PAX8 is as a bona fide marker of fimbrial-originated tumors. This regulatory transcription factor plays a key role in the development of the nervous system and embryogenesis of the renal/upper urinary tract, Müllerian duct, and the thyroid. Moreover, PAX8 is a specific marker for these tumors, suggesting an important role in tumor development^75^. However, the cell of origin that undergoes malignant transformation in ovarian carcinomas has not yet been identified, and the gathered pieces of evidence could not, until now, satisfactorily explain the histopathological heterogeneity of this malignancy.

Interestingly, we found an atypical cell type in our ovarian carcinoma cultures, which we called “twin” cells. This cell type has a distinctive neuronal and weak or absent epithelial signature, at least at the single-cell transcriptional level. These cells also exhibit stemness properties like the expression of the key transcription factors Klf-4, SOX2 and Nanog, among others. Curiously, its dark color suggested that they produce neuromelanin (NM), an aspect first addressed using the Fontana-Masson staining which is specific for the histological detection of this pigment and secondly confirmed by the high expression of Tyrosine hydroxylase (TH), a key enzyme in the generation of this pigment, a sub-product of catecholamine metabolism. Interestingly, TH was found enhanced in cisplatin sublines. This observation, together with the neuronal background present in ovarian carcinoma cells as demonstrated at transcriptional and protein levels, lead to the conclusion that these cells have a clearly ectodermal origin, similar to melanocytes.

Investigating ovarian carcinoma cells obtained from fresh ascites (e.g. OC236 cells), we observed a population of cells with neuronal/stemness signature, accompanied by the expression of ABC transporters, which are widely expressed by stem cells^76,77^ as tool for chemoprotection and detoxification. This signature confirms the nature of the cells from which ovarian carcinoma tumors evolve.

We did not restrict our study to cell-based models and therefore examined the existence of similar cells in paraffin sections from ovarian tissues. The Fontana-Masson staining revealed the presence of melanin-producing cells in both regular ovarian as well as in the ovarian carcinoma tissues. This finding suggests that some resident cells in the normal ovary may possess neuronal or neuroepithelial characteristics, which persist in ovarian carcinoma tissues. The global gene expression profile of SKOV3^CP^ aligns with a phenotype characteristic of neuroepithelial and radial glial cells. These cells are considered the progenitors capable of generating post-mitotic neurons (ectodermal), astrocytes and oligodendrocytes (mesodermal), with Notch1 and SOX2 playing an important role in these processes. Glial radial cells are anatomically localized to the ventricular zone, a zone considered to be where neurogenesis occurs^78^. If this theoretical “ovarian-annex resident neuronal lineage cell” has the full quality of a neuron or its precursors, how do they gain the epithelial condition? One of the first possibilities is inferred from this work: from neuronal to epithelial (the reverse process evidenced by cisplatin activity on ovarian cancer cells towards neuronal) by triggering backward differentiation due to harsh environments^79,80^. Another hypothetical possibility is via viral infection, where HERV envelope proteins with fusogenic potential are transactivated by a neurotropic virus and their possible helpers (given the neuronal background of ovarian carcinoma cells) or epigenetic stress. This induces cell–cell fusion with adjacent epithelial cells and the formation of heterokaryons with different epigenetics, and therefore genome instability, which is the very first event in oncogenesis. Cell–cell fusion events, moreover, are widely observed in 2D cultures in ovarian carcinoma cells. In this regard, transcriptome analysis focused on HERVs expression in ovarian carcinoma cells revealed a vast expression of envelope proteins with fusogenic potential. Among wildtype cells, HERV-H, a paleovirus several times integrated in our genome in a relatively intact form, is the one most expressed. Additionally, in cells exposed to cisplatin, Syncytin 1 (HERV-W_E1_), Syncytin 2 (HERV-FRD_1_), HERV-K, HERV 3.1 and HERV-P_env_ and other envelope proteins of retroviral elements were found to be overexpressed, suggesting the gain of fusogenic capacity of this sub-cell line.

Eager to distinguish and localize the neuronal or neuroepithelial cell type that might be implied in the tumorigenesis in ovarian tissues, we sought to search for cells with such a molecular signature in both tissues, regular and tumoral. We found that there are some cell populations in “regular” (non-tumoral) ovarian tissues double positive for KRT80 and ChgA, the latter being a specific marker for neuronal or neuroendocrine cells. Whereas those cells were rare in “regular” ovarian tissues, we found a huge amplification of those cell populations in ovarian carcinoma tissues, indicating a possible expansion of those populations during tumor evolution.

Of note, patients suffering from different types of cancer, develop an antibody-mediated immune response triggered by the PNMA1 antigen, a 37-kDa protein restricted to neurons, testis cells and cancer cells. These anti-PNMA1-autoantibodies cross-react with the nervous system structures inducing vast neurological impairments like limbic and extra- limbic encephalopathies, Bergmann gliosis, cerebellar ataxia, etc.^81^. The ovarian cell lines studied in this work, express PNMA1, which is enhanced in cisplatin-exposed cells. Decisively, we also found that those cell populations in “regular” (non-tumoral) ovarian tissues, which are dually PNMA1-ChgA positive, are exactly the cell populations that are expanded to form the tumor bulk. However, we have not addressed the character or physiology of this cell type.

This relevant observation resulting from transcriptome analysis clearly indicates the neuronal or neuroepithelial origin of some ovarian cancers and possibly of several tumor types. This neuronal or epithelial signature of ovarian carcinomas is raised from a rare cell type found in regular (non-tumoral) ovarian tissues which have a particular phenotype expressing Tyrosine hydroxylase, ChgA, the paraneoplastic protein PNMA1 and KRT80. In this regard, perhaps we have to reconsider a possible tissue-resident cell type remnant from the neural crest cells that undergo malignant transformation (e.g. neuroblastoma^82^) as a cell of origin in this tumor type and possibly others of epithelial histology and a neuronal background. Although we did not address the physiological role of this cell type, it is very likely that those resident cells represent a kind of progenitor cells with strong neuronal features. Furthermore, a closer examination of the media compositions used for generating and culturing tumor-derived organoids reveals that nearly all, if not all, contain cytokines and factors that promote neuronal growth in vitro.

The triad of phenotype, transcriptome and protein expression in mesoderm-derived ovarian carcinoma cells undergoes a neuronal-like differentiation upon cisplatin exposure. Although the neuronal phenotype remains for several weeks, this condition is gradually reversed over time, with the cells regaining their epithelial morphology. However, it remains unclear whether the acquisition of neuroepithelial features in OC corresponds to more aggressive tumor types or persistent chemotherapy refraction of dormant OC cells^83^. Additionally, the transformation of ovarian carcinoma cells into neuron-like cells induced by platinum-based drugs does not result in fully functional neurons, as the cells exhibit no significant electrophysiological activity typical of differentiated neurons. This indicates that these cells maintain an undifferentiated phenotypical status. The factor impeding complete differentiation of ovarian carcinoma cells into mature neurons may reside in the efficiency of chemically defined media to recap the physiological conditions and the microenvironment of neuronal tissues.

It is important to note that cisplatin only slightly enhances the neuronal characteristics that ovarian carcinoma cells naturally possess. Cisplatin induces EMET, which is strongly associated with resistance. Furthermore, we are not addressing the anatomical origin of ovarian carcinomas but rather the type of cell from which this tumor evolves. Whether these cells migrate from the fimbria to the surface of the ovary or vice versa requires further investigation. In this regard, we have to consider the highly fusogenic activity of cells during malignant transformation, a fact that might render in heterokaryons containing different epigenetics from the original fused cells and therefore this could be a confounder from the histological point of view. For example, a transformed X cell from the ovary surface fuses with the proximal cells in the fimbria (two cells of different epigenetics) resulting in an admixture in which cells might acquire a new phenotype and the morphology of the predominant cells (e.g.: fimbria?).

In summary, platinum-based cytostatics force epithelial ovarian cancer cells into a phenotype resembling neural crest cells, which are progenitors for various cell lineages, including radial glial cells. This transformation indicates a mesodermal to ectodermal transition, or an "epithelial-mesenchymal-ectodermal transition" (EMET). Finally, an ovarian resident cell type expressing TH, Neuromelanin, PNMA1, KRT80, and ChgA appears to be the cell type that undergoes expansion during carcinogenesis.

## Electronic supplementary material

Below is the link to the electronic supplementary material.

Supplementary Material 1

Supplementary Material 2

Supplementary Material 3

Supplementary Material 4

Supplementary Material 5

Supplementary Material 6

Supplementary Material 7

## Data Availability

All data supporting this study as well as primary cells used are reasonably available upon request. Transcriptome data from this study was deposited in NCBI GEO under the accession number GSE264296.
