# Supplementary material for: The neuroepithelial origin of ovarian carcinomas explained through an epithelial-mesenchymal-ectodermal transition enhanced by cisplatin
Source: Sci Rep. 2024 Nov 26;14:29286. doi: 10.1038/s41598-024-76984-9 (PMC11599565; doi:10.1038/s41598-024-76984-9)
Supplement: Supplementary file 6 — Supplementary Material 6 [file 41598_2024_76984_MOESM6_ESM.docx]

**Table ST1**: Medium composition for the generation ovarian carcinoma organoids

| **Components** | **Final conc** |
| --- | --- |
| L-glutamine | 2 mM |
| Penincilin/Streptomycin/Amphotericin B (100x) | 1x |
| A83-01 | 0,25 µM |
| Nicotinamide | 5 mM |
| N-acetylcysteine | 1,25 mM |
| N2 (100x) | 1x |
| B27 minus vitamin A (100x) | 1x |
| 17-ß Estradiol | 10 mM |
| p38i (SB203580) | 1 µM |
| EGF | 50 ng/ml |
| Prostaglandin E2 | 10 nM |
| Noggin | 100 ng/ml |
| RPON1 | 50 ng/ml |
| IGF1 | 20 ng/ml |
| HGF | 10 ng/ml |
| NRG1 | 50 ng/ml |
| HEPES | 10 mM |
| Y27632 (RI) | 10 µM |

**Table ST2**: List of antibodies used for ICC, IHC, FACS and Western blot analysis

| **Antibody** | **Clonality** | **Dilution** | **Host** | **Cat No** | **Manufacturer** |
| --- | --- | --- | --- | --- | --- |
| Actin | Monoclonal | 1:100 | Rabbit | A2066 | Sigma Aldrich |
| ALDH1A1 | Monoclonal | 1:100 | Rabbit | #36671 | Cell Signaling |
| Beta III Tubulin | Monoclonal | 1:100 | Mouse | #4466 | Cell Signaling |
| Beta III Tubulin | Monoclonal | 1:100 | Rabbit | #4460 | Cell Signaling |
| Beta Tubulin | Monoclonal | 1:100 | Mouse | Sc-55529 | Santa Cruz |
| CD44 | Monoclonal | 1:100 | Mouse | #3570 | Cell Signaling |
| Chromogranin A | Monoclonal | 1:5000 | Mouse | A3 | DAKO |
| Chromogranin A | Polyclonal | 1:2000 | Rabbit | 10529-1-AP | Proteintech |
| Doublecortin | Monoclonal | 1:100 | Rabbit | #4604 | Cell Signaling |
| E-cadherin | Monoclonal | 1:100 | Rabbit | #3195 | Cell Signaling |
| EpCAM | Monoclonal | 1:100 | Mouse | #93790 | Cell Signaling |
| EpCAM (VioBlue) |  | 1:50 | Recombinant | 130-111-004 | Miltenyi Biotech |
| EpCAM | Polyclonal | 1:2000 | Rabbit | 21050-1-AP | Proteintech |
| FABP7 | Monoclonal | 1:100 | Rabbit | #13347 | Cell Signaling |
| Goosecoid | Polyclonal | 1:100 | Goat | AF4086 | R&D Systems |
| HES1 | Monoclonal | 1:100 | Rabbit | #11988 | Cell Signaling |
| KRT80 | Polyclonal | 1:500 | Rabbit | 16835-1-AP | Proteintech |
| LEF1 | Polyclonal | 1:100 | Goat | #2230 | R&D Systems |
| MAP2 | Monoclonal | 1:100 | Rabbit | #4542 | Cell Signaling |
| N-cadherin | Polyclonal | 1:100 | Rabbit | #13116 | Cell Signaling |
| NeuN | Monoclonal | 1:100 | Rabbit | #12943 | Cell Signaling |
| NeuroD | Monoclonal | 1:100 | Rabbit | #4373 | Cell Signaling |
| Neurofilament-L | Monoclonal | 1:100 | Rabbit | #2837 | Cell Signaling |
| Notch1 | Monoclonal | 1:100 | Rabbit | #4530 | Cell Signaling |
| Notch3 | Polyclonal | 1:2000 | Rabbit | 55114-1-AP | Proteintech |
| Occludin | Monoclonal | 1:100 | Mouse | MAB7074 | R&D Systems |
| Oct4 | Polyclonal | 1:100 | Mouse | 60242-1-AP | Proteintech |
| PAX6 | Polyclonal | 1:100 | Sheep | AF8150 | R&D Systems |
| PAX6 | Polyclonal | 1:2000 | Rabbit | 12323-1-AP | Proteintech |
| PAX8 | Polyclonal | 1:2000 | Rabbit | 10336-1-AP | Proteintech |
| PAX9 | Polyclonal | 1:1000 | Rabbit | PAH449Hu01 | Cloud Clone Corp. |
| PNMA1 | Polyclonal | 1:500 | Rabbit | 13631-1-AP | Proteintech |
| PSD95 | Monoclonal | 1:100 | Rabbit | #3450 | Cell Signaling |
| Slug | Monoclonal | 1:100 | Rabbit | #9585 | Cell Signaling |
| Snail | Polyclonal | 1:100 | Goat | AF3639 | R&D Systems |
| SOX2 | Monoclonal | 1:100 | Rabbit | #4900 | Cell Signaling |
| Stathmin | Monoclonal | 1:100 | Rabbit | #13655 | Cell Signaling |
| Synaptophysin | Monoclonal | 1:100 | Rabbit | #5467 | Cell Signaling |
| Tenascin C | Monoclonal | 1:100 | Rabbit | #12221 | Cell signaling |
| TH | Polyclonal | 1:500 | Rabbit | 25859-1-AP | Proteintech |
| Vimentin | Monoclonal | 1:100 | Rabbit | #5741 | Cell Signaling |
| Anti-β-Actin-HRP | Monoclonal | 1:50000 | Rabbit | A3854 | Sigma Aldrich |
| Anti-Rabbit IgG Alexa 555 | Polyclonal | 1:1000 | Goat | #4413 | Cell Signaling |
| Anti-Mouse IgG Alexa 488 | Polyclonal | 1:1000 | Goat | #4408 | Cell Signaling |
| Anti-Sheep IgG NL-557 | Polyclonal | 1:1000 | Donkey | NL010 | R&D Systems |
| Anti-Rabbit-HRP IgG | - | 1:1000 | Goat | #7074 | Cell Signaling |
| Anti-Mouse-HRP IgG | - | 1:1000 | Goat | #7076 | Cell Signaling |
| Anti-Rabbit IgG Alexa 488 | Polyclonal | 1:1000 | Goat | #4412 | Cell Signaling |
| Anti-Mouse IgG Alexa 555 | Polyclonal | 1:1000 | Goat | #4409 | Cell Signaling |
| Anti-goat-HRP IgG | Polyclonal | 1:1000 | Donkey | Sc-2020 | Santa Cruz |
| Anti-goat-FITC IgG | Polyclonal | 1:1000 | Rabbit | ABIN101210 | Antibodies-online |
| Anti-goat-Alexa 568 | Polyclonal | 1:1000 | Donkey | Ab175704 | Abcam |

**Table ST3**: Name and UniProt ID of the gene studied.

|  | **Name** | **UniProt Protein Name** | **UniProt  Gene name** | **UniProt**  **Entry Nr.** |
| --- | --- | --- | --- | --- |
| 1 | ACHE | Acetylcholine receptor subunit epsilon | ACHE | Q04844 |
| 2 | ALDH1L1 | Cytosolic 10-formyltetrahydrofolate dehydrogenase | ALDH1L1 | O75891 |
| 3 | Ascl1 | Achaete-scute homolog 1 | ASCL1 | P50553 |
| 4 | BDNF | Brain-derived neurotrophic factor | BDNF | P23560 |
| 5 | CD24 | Signal transducer CD24 | CD24 | P25063 |
| 6 | E-Cadherin | Cadherin-1 | CDH1 | P12830 |
| 7 | N-Cadherin | Cadherin-2 | CDH2 | P19022 |
| 8 | CHAT | Choline O-acetyltransferase | CHAT | P28329 |
| 9 | Doublecortin | Neuronal migration protein doublecortin | DCX | O43602 |
| 10 | PSD95 | Disks large homolog 4 | DLG4 | P78352 |
| 11 | Ephrin B1 | Ephrin-B1 | EFNB1 | P98172 |
| 12 | TBR2 | Eomesodermin homolog | EOMES | O95936 |
| 13 | EpCAM | Epithelial cell adhesion molecule | EPCAM | P16422 |
| 14 | GAD67 | Glutamate decarboxylase 1 | GAD1 | Q99259 |
| 15 | GDNF | Glial cell line-derived neurotrophic factor | GDNF | P39905 |
| 16 | NMDAR1 | Glutamate receptor ionotropic, NMDA 1 | GRIN1 | Q05586 |
| 17 | NMDAR2B | Glutamate receptor ionotropic, NMDA 2B | GRIN2B | Q13224 |
| 18 | Goosecoid | Homeobox protein goosecoid | GSC | P56915 |
| 19 | HES1 | Transcription factor HES-1 | HES1 | Q14469 |
| 20 | HES5 | Transcription factor HES-5 | HES5 | Q5TA89 |
| 21 | HIF1 alpha | Hypoxia-inducible factor 1-alpha | HIF1A | Q16665 |
| 22 | Klf-4 | Krueppel-like factor 4 | KLF4 | O43474 |
| 23 | Map2 | Methionine aminopeptidase 2 | MAP2 | P50579 |
| 24 | c-Myc | Myc proto-oncogene protein | MYC | P01106 |
| 25 | Nanog | Homeobox protein NANOG | NANOG | Q9H9S0 |
| 26 | Nestin | Nestin | NES | P48681 |
| 27 | NDF1 | Neurogenic differentiation factor 1 | NEUROD1 | Q13562 |
| 28 | Neurog2 | Neurogenin-2 | NEUROG2 | Q9H2A3 |
| 29 | NGF | Beta-nerve growth factor | NGF | P01138 |
| 30 | Noggin | Noggin | NOG | Q13253 |
| 31 | Notch1 | Neurogenic locus notch homolog protein 1 | NOTCH1 | P46531 |
| 32 | Nurr1 | Nuclear receptor subfamily 4 group A member 2 | NR4A2 | P43354 |
| 33 | Neurturin | Neurturin | NRTN | Q99748 |
| 34 | Occludin | Occludin | OCLN | Q16625 |
| 35 | PAX6 | Paired box protein Pax-6 | PAX6 | P26367 |
| 36 | Oct-4 | POU domain, class 5, transcription factor 1 | POU5F1 | Q01860 |
| 37 | CD133 | Prominin-1 | PROM1 | O43490 |
| 38 | Sgne-1/SCG5 | Neuroendocrine protein 7B2 | SCG5 | P05408 |
| 39 | Snail | Zinc finger protein SNAI1 | SNAI1 | O95863 |
| 40 | Slug | Zinc finger protein SNAI2 | SNAI2 | O43623 |
| 41 | Sox2 | Transcription factor SOX-2 | SOX2 | P48431 |
| 42 | Sox9 | Transcription factor SOX-9 | SOX9 | P48436 |
| 43 | Stathmin | Stathmin | STMN1 | P16949 |
| 44 | Syne-1 | Nesprin-1 | SYNE1 | Q8NF91 |
| 45 | Synaptophysin | Synaptophysin | SYP | P08247 |
| 46 | TBR1 | T-box brain protein 1 | TBR1 | Q16650 |
| 47 | Tenascin C | Tenascin | TNC | P24821 |
| 48 | Beta III tubulin | Tubulin beta-3 chain | TUBB3 | Q13509 |
| 49 | Twist 1 | Twist-related protein 1 | TWIST1 | Q15672 |
| 50 | Vimentin | Vimentin | VIM | P08670 |
| 51 | ZEB1 | Zinc finger E-box-binding homeobox 1 | ZEB1 | P37275 |

**Table ST4**: Primer and probe sequences for qPCR designed with IDT PrimerQuest^TM^ Tool. All primers and probes are reflected in 5´→3´ polarity

| **Name** | **Forward** | **Probe** | **Reverse** |
| --- | --- | --- | --- |
| 18S  (NR_003286) | GGACATCTAAGGCATCACAG | TGCTCAATCTCGGGTGGCTGAA | GAGACTCTGGCATGCTAACTAG |
| ACHE  (M55040.1) | CTCAACGTGTGGACACCATA | CCCTGTCCTCGTCTGGATCTATGGG | CTCCTTGGACGTGTACGATG |
| β III Tubulin  **(**NM_006086.3) | GGGCCAAGTTCTGGGAAG | TCAGTGATGAGCATGGCATCGACC | GGATCAGCGTCTACTACAACG |
| Choline O-acetyltransferase  (NM_001142934.1) | AAAGGCCAGCTGTCAGG | ACTATGGGCTCTTCTCCTCCTACCG | CATACCCAAGGACACGCTG |
| Doublecortin  (AJ003112.1) | CTGTGTCCTCTGACCGTTT | TGACCTGACGCGATCTCTGTCTGA | CTCAGGGAGTGCGTTACATT |
| E-cadherin  (Z13009.1) | CAGAAGACAGAAGAGAGACTGG | GTTATTCCTCCCATCAGCTGCCCA | TCCAACAAAGACAAAGAAGGC |
| FABP7  (NM_001319041.1) | GATGAGTACATGAAGGCTCTAGG | TAGGCAGGTGGGAAATGTGACCAA | AGAAGGAGACAAAGTGGTCATC |
| GAD65  (M81882.1) | CTTTCGGGTCGGAAGATGG | CCAAGTGGCGCAGAAGTTCACGGG | CATCGGAAACAAACTGTGCG |
| GFAP  (AF419299.1) | CTTTGCCAGCTACATCGAGAA | GGTTCGCTTCCTGGAACAGCAAA | CAAGGAGCCCACCAAGC |
| HES1  (Y07572.1) | GGGAAAGATTGCAAGGTGAATAAA | CGTGTCCTGAAGGAGTTCCACCAG | CTTGTGCTGCATTGCACC |
| HES5  (DQ272660.1) | CAGCATCGAGCAGCTGAA | CTGAAGCACAGCAAAGCCTTCGTC | TACAGCGAAGGCTACTCGT |
| MAP2  (NM_002374.3) | GAAGATAAATCAGGAATGTCCAAG | ACATCTGCCTTGAAAGAAGAAGCAACA | AGGCAGTGATTCTATGAACTGA |
| N-cadherin  (X57548.1) | CTTCATGCCGGTACCATGT | ACAACATTCACTGCTCAGGACCCA | AAATTATCTGATCCTGCCAATTGG |
| NDF1  (AB593070.1) | GAAGAGGAGGATGACGATCAAA | AGAAGATGACTAAGGCTCGCCTGGA | ATTGAGACGCATGAAGGCT |
| NMDAR1  (NM_007327.3) | GATGGCTCTGT CGGTGTG | AGGTCTACGCCA TCCTAGTTAGCCA | CCAACGACCAC TTCACTCC |
| NMDAR2B  (U88963.1) | GATGCCCACGA GAAAGATGA | AGCCATGAATGA GACCGACCCAAA | CATCATCACC CGCATCTGT |
| Nurr1  (NM_006186.3) | CCAGCTTCAGTA CCTTTATGGA | AGCCACCTTGCTTGTACCAAATGC | CAGCAGTCCTCCATTAAGGTAG |
| Occludin  (NM_002538.3) | GCAATGACATATATGGTGGAGAGA | GTTCGACCAATGCTCTCTCAGCCA | TACAAATGGACCTCTCCTCCA |
| Pax6  (NM_000280.4) | AGGCTCAAATGCGACTTCA | GCTGAAGCGGAAGCTGCAAAGAAA | GAGAAAGAGTTTGAGAGAACCC |
| PSD95  (U83192.1) | GTGCTGCCCACAAGGAT | GGGAGGTTGCAGATTGGAGACAAGA | TGAAGATGCTGTGGCAGC |
| Stathmin  (X53305.1) | GAGCACGAGAAAGAAGTGCT | GAAGGCAATAGAAGAGAACAACAACTTC | CAGAAGAGAAACTGACCCACAA |
| Synaptophysin  (NM_003179.2) | CAGGCTGCACCAAGTGTA | GGGCACCACCAAGGTCTTCTTAGTT | GTCAGCCGAATTCTTTGTCAC |
| TBR2  (NM_001278182.1) | ACCAATAACAAAGGCGCAAATA | ACAACACCCAGATGATAGTCTTACAATCC | CATATTGTTGAAGTTACAGAGGA |
| Tenascin-C  (NM_002160.3) | CATCTGTGACGACGGCTT | CAATGACCAGGGCAAGTGCGTAAA | GGAGTCTGCATC TGTTTCGA |
| NeuN / RBFOX3  (NM_001082575.3) | ACTACTCCGGCCAGACC | ATGGCATGACCCTGTACACACCAG | CGATGGGCTGTGTGCTG |
| Vimentin  (NM_003380.5) | CTCAATGTCAAGGGCCATCT | ACGAAGGTGACGAGCCATTTCCTC | GCTGCTAACTACCAAGACACTAT |
| Notch1  (NM_017617.5) | CAGTGTCGAGATGGCTATGA | TGTGTGTTACCTACCACAATGGCACA | CCTTCTGGACATTTGCAGTATC |
| Nanog  (NM_024865.4) | CAGGACAGCCCTGATTCTTC | CAGTCCCAAAGGCAAACAACCCAC | GTTTCTTGACCGGGACCTT |
| KLF4  (NM_001314052.2) | AGGAGCCCAAGCCAAAG | TAATCACAAGTGTGGGTGGCGGTC | GCCTTGAGATGGGAACTCTT |
| SOX2  (NM_003106.4) | CGGACAGCGAACTGGAG | AGAGGAGAGTAAGAAACAGCATGGAGA | TTTGAGCGTACCGGGTTT |
| Oct-4 / POU5F1  (NM_002701.6) | GCTTAGCTTCAAGAACATGTGTA | TGGGTGGAGGAAGCTGACAACAAT | CACGAGGGTTTCTGCTTTG |
| CD24  (NM_001291737.1) | CAAATCCAACTAATGCCACCAC | TCGTGGTCTCACTCTCTCTTCTGCA | GTTTCTTGGCCTGAGTCTCTT |
| CD44  (NM_000610.4) | AATGGCCCAGATGGAGAAAG | TGAGCATCGGATTTGAGACCTGCA | TTGCTGCACAGATGGAGTT |
| EpCAM  (NM_002354.3) | GGTGATGAAGGCAGAAATGAATG | CCCATCATTGTTCTGGAGGGCC | TCATCGCAGTCAGGATCATAAAG |
| Tyrosine hydroxilase  (NM_199292.3) | TGTACTGGAAGGCGATCTCA | AAGTTCGACCCTGACCTGGACTTG | GAGCTGGACAAGTGTCATCA |
| PNMA1  (NM_006029.5) | CCACTCCTCTAGGACCTCATTA | ATCAAAGGTTTCCTCTCCAGGCCC | GGTACAAGAGGCTGACACTTT |
| KRT80  (NM_001081492.2) | TTCGAAACTCCTCAACCTTCTC | AGGAGGAACTGCGCAAAGTGAGC | CGGGCATCTCTATGAGGAATATC |
| HERV-IDDMK  (AF012337.1) | CACCAGTCACATGGATGGATAA | AATGATAGTGTATGGGTACCTGGCCC | CCCTTCTTCCTCAGGTTTGG |
| HERV-Wenv  (AF072506.2) | GTGCCTGGGTTTGTTCTAATTG | AAGCCGTGGG CACAGAAGAGAAC | ATCTTGGGCCATGTGGTAAG |
| HERV-Wprotease  (FN806835.1) | TGAAGACTGGGCAACAGAAG | TTGAACAGGACGGGCATTCTTTGC | GGTACTTCCTTTGGTAGGGAAAG |
| HERV-K seminoma  (JN675049.1) | GTAGAGAGCCACCTGACTTATG | ACATTGGGCTAGTCAATGTCGTTCT | TG CTCGTTTCCCGACAAT |
| ERV-MER-34-1  (XR_941039.1) | CGAGCCTTCTTCTTGGTCTT | TTTATCCCTCGGAGTTGCACTGGC | CCGAAACCCGCTACAAAGTA |
| ERV-V1  (NM_152473.2) | GACACCAGTGAGTCAAGTTAG G | CCACCATCCATCCCATGAGCTTCT | GTGACAGGAAGAACACCTCTG |
| ERV-V2  (NM_001191055.2) | CCAAGAAGACCAAGAACCAGAG | CCTGGTTACCTCGCCTGGTTATCC | CTGGAGATGCCAGAAATGTTG |
| HERV-3.1  (NM_001007253.3) | GTAGTCTTCACTGCTCTGTGTC | CTTCTGTGGCCTCGTTACCTGCA | CTTAGCTGTGATCTCCCAATAC |
| HERV-FRD1  (NM_207582.2) | AAACACCAG G G ACAG CTTATC | TCG CCCAG AG AATG G ACAAG CAT | G CCTCATCAGTCCTTTCAG ATT |
| HERV WE1  (NM_001130925) | GAGTTCAAGATCAGGCAAGAGA | AAGAAGTAATCTCCCAACTCACCCGG | GTACGGAGGGTTTCATGTAGTT |
| HERV-C  (AB266802.1) | GCTACCTTGCTCCCAGTATTAG | TCCCTGCGAGCATGACTGTGTAGA | CATCCACGTATAGTTCCCAGTC |
| HERV-Tenv  (AB120695.1) | CCAGAAGGACGACAACTCATC | TGAGTTACATCCCAGGTTGCACCA | GCTATGCTAAGACCAGCCAATA |
| HERV-Spol  (AB162179.1) | AGGAGCTGCAAATAGTGAGTG | TCAGAAAGCCTGATGGGACTTGGC | TGCTTCATTCAGTTCCCGATAA |
| HERV-Fpol  (AB120695.1) | GATGCTAACATTCAGCCCAAATAC | TCTACATCTCCTCTCCTCCCAAGCA | GGGAAGGAGGAGGAGAACTTA |
| HERV-L  (EF141078.1) | GACTAGTAGGGTGAGGGCTATTA | AAGCCATTTGAGCTGCCTCTACCT | CACCAGGGATGCAGTATTGT |
| HERV-Kenv  (DQ069916.1) | AGTCTTTCTACCCTTGGGAATG | TGGATGTTCAGGACCAGAAACAGGAC | GCCACAGTAAGCCTCCATAA |
| HERV-Kprt  (DQ159119.1) | GCGAAATTCAGCTGGTGATTAG | CGATCGTATTGCGCAGCTGCTG | GCTGTTGCCGCCTTTAATATAC |
| HERV-Kpol  (Y10391.1) | CACCAGTCACATGG ATG G ATAA | CAGGGCAGCGATCATCTATGGGTC | CCCTTCTTCCTCAGGTTTG G |
| HERV-Kgag  (DQ157733.1) | GGCCAGTATTGATAGGCTACTG | CACAAGTCCTCTTCCAGCAGGTCA | CCATCAGAGTCTAAACCACGAG |
| HERV-KISD  (DQ360584.1) | CAGTCAGTAAACTTTGTTAATGATTGGC | TGATCTTAGACAAACTGTCATTTGGATGGG | TGTTCTAAGCTCATGAGTCTGTCT |
| HERV-KLTR  (AB047320.1) | AAATATGGCCTCGTGGGAAG | CCCATAAAGGGTCTGTAACTGAGGAGGA | CCTCTTGTCTCAACTGCAAG A |
| HERV-Penv  (AB240043.1) | AGGCCTCTGAGGTAGTAAAGA | AAAGTGCCTCCAAAGTGAAATGGCC | GTGATGGCTGCTTCAAATGAG |
| HERV-Pgag  (AB240024.1) | CCAGTGTTATCCAAAGG CTTCTA | TGCCAGCAACAGG ATCTCCAACTT | GGAGATATGGTGGTTGCAGATAG |
| HERV-Ppol  (AB240030.1) | CTCTCAATACCTGTCTGCCTTC | TG CCTCTGACCAAACCATCCAGTT | AATGCCTGTCCAAACAAATGAA |
| HERV-PG  (AF081551.1) | CACCCAATCGGAGGTATCTTG | TGGTCAGGACATTCCAAGGCAACA | CCACGGAGGTCATGAAGTATT |
| HERV-E  (AB259286.1) | GATG GCTTAATGGTGCCAATG | TACCTGATCAATGGGCAGGCAGTT | ACGGCTCCAGCTAGACTATA |
| HERV-FC1  (AJ507128.1) | GCCTCATCAGTCCTTTCAGATT | TCGCCCAGAGAATGGACAAGCAT | AAACACCAGGGACAGCTTATC |
| HERV-HC2pol  (AB167348.1) | GTGAATCCTTCCTTCTGGAACT | CAGGGAGCACACTCCAATCCAGTT | CCAACAGGTATGAGACACCATC |
| HERV-Henv  (AH007767.2) | CAGTATTGATAGAGGGTCTGTCTG | CCGAAGCCCAACTACACACATCACT | GGTAATCTTTCACCTTCTCGATGT |

**Figure SF1**: **Dose-Response curve of SKOV3 cells treated with cisplatin**. Using MTT proliferation assay the IC_50_ for cisplatin in SKOV3^WT^ is approximatelly 2.1 μg/mL and the SKOV3^CP^ cells is 11.7 μg/mL. The value obtained for SKOV3^WT^ is in agreement with several publications (for consulting this paper: <https://doi.org/10.3892/ijo.2017.4023>)

OVCAR-3^CP^ 10 days exposure

OVCAR-3^WT^

OVCAR-3^CP^ 6 days exposure

OVCAR-3^CP^ 8 days exposure

**Figure SF2: Gain of neuronal morphology of OVCAR-3 ovarian carcinoma cells after cisplatin exposure**. Changes in OVCAR-3 ovarian carcinoma cells is observable like in SKOV3 cells. The cell morphology upon platinum-derived drug exposure enforce a neuronal-like shape of cells.

Colon Normal

(Pat. 4)

Neuronal formation

Neuropan Medium

Colon Normal

(Pat. 19)

Neuronal formation

Neuropan Medium

Colon Normal (Pat. 114)

Neuronal formation

Neuropan Medium

**Figure SF3A**: **Neuronal differentiation of human colon normal cells**. Cells isolated from normal colon tissues were cultivated in Neuropan medium for two weeks. The neuronal morphology is reached in the same way as described for rat brain cells, ovarian carcinoma cell lines or cells isolated from ascites. Arrows indicate cells in process of neuronal differentiation.

OC Patient 28^CPT^ Ascites

OC Patient 28^CPT^ Ascites

**Figure SF3B**: **Development of dendrite-like structures in DMEM-cultured cells obtained from ovarian carcinoma ascites**. Ovarian carcinoma cells from heavily PIPAC-treated patients were cultured in DMEM. A progressive cytoplasm shrinkage takes place and is the precursor event of dendritic trees development. The development of neuronal structures reminiscent of multipolar neurons is clearly analogous to normal neuron development observed in brain cells isolated from rats as well as in SKOV3^CP^ cell subline. Cells in different stages of the neuronal morphological remodeling are depicted.

**Figure SF4**: Organoids growth kinetic from different OC cells

**SKOV3^WT^**

3D day 1, 40×

Ø treatment

**SKOV3^WT^**

3D day 1, 40×

2 µg/mL cisplatin

**SKOV3^WT^**

3D day 1, 40×

1 µM MAPRK4-I

**SKOV3^WT^**

3D day 2, 40×

Ø treatment

**SKOV3^WT^**

3D day 2, 40×

2 µg/mL cisplatin

**SKOV3^WT^**

3D day 2, 40×

1 µM MAPRK4-I

**SKOV3^WT^**

3D day 5, 40×

Ø treatment

**SKOV3^WT^**

3D day 5, 40×

2 µg/mL cisplatin

**SKOV3^WT^**

3D day 5, 40×

1 µM MAPRK4-I

**SKOV3^WT^**

3D day 6, 40×

Ø treatment

**SKOV3^WT^**

3D day 6, 40×

2 µg/mL cisplatin

**SKOV3^WT^**

3D day 6, 40×

1 µM MAPRK4-I

**SKOV3^WT^**

3D day 7, 40×

Ø treatment

**SKOV3^WT^**

3D day 7, 40×

2 µg/mL cisplatin

**SKOV3^WT^**

3D day 7, 40×

1 µM MAPRK4-I

**SKOV3^WT^**

3D day 8, 40×

Ø treatment

**SKOV3^WT^**

3D day 8, 40×

2 µg/mL cisplatin

**SKOV3^WT^**

3D day 8, 40×

1 µM MAPRK4-I

**OC236^WT^**

3D day 1, 40×

Ø treatment

**OC236^WT^**

3D day 4, 40×

Ø treatment

**OC236^WT^**

3D day 5, 40×

Ø treatment

**OC236^WT^**

3D day 6, 40×

Ø treatment

**OC236^WT^**

3D day 7, 40×

Ø treatment

**Figure SF4**: Generation of OC organoids from SKOV3, OVCAR-3 and the primary OC cells. During the organoid differentiation, specifically within the first days of culture, prominent neuronal-like populations is observed. Culturing conditions are reflected in the main document.

OC Patient 28^CPT^ Ascites

OC Patient 28^CPT^ Ascites

**Figure SF5**: Presence of “Twin” cells in patient material. In ovarian carcinoma cells isolated from PIPAC-treated patients and cultured in DMEM is possible to observe cells, which resemble “Twin” cells as in the SKOV3 model.

OC Patient 10^CPT^ Ascites

OC Patient 69^CPT^ Ascites

OC Patient 60^CPT^ Ascites

OC Patient 21^CPT^ Ascites

**Figure SF6**: **Twin and** n**euronal-like cells found in ascites from patients treated with platinum compounds**. PIPAC (pressurized intraperitoneal aerosol chemotherapy) is an intervention conceived for unresectable platinum resistant ovarian cancer (<http://dx.doi.org/10.1136/ijgc-2019-IGCS.56>). Ascites cells obtained from patients previously treated with platinum compounds using PIPAC were isolated and cultivated in DMEM. Some cells show characteristic neuronal morphology. Pictures were obtained from our bank in a random way. CPT: cisplatin treated.

**Figure SF7**: Genes related to the cardiac and skeletal muscle physiology found in SKOV3 cells, wild type and expossed to cisplatin for 72 hours.

I

II

**Figure SF8**: Transcriptional expression of Human Endogenous Retroviruses (HERVs) in ovarian cancer cell lines **I**. Cisplatin repress several HERV genes normally found regularly in SKOV3^WT^. However, some of these retroviral elements were found upregulated after short exposure to cisplatin, among them HERV-WE1 (Syncytin 1), HERV-FRD1 (Syncytin 2) and HERV-Wenv. **II**: qPCR screening for HERV elements in OC236^WT^ cells.

**Vimentin WT-0.26%**

**Vimentin CP-96.26%**

**βIII-Tubulin WT-0.52%**

**βIII-Tubulin CP-9.02%**

**WT-0.52%	CP-9.02%**

**DCX WT-29.54%**

**DCX CP-96.35%**

**WT-29.54% CP- 96.35%**

**E-Cadherin WT-89.92%**

**E-Cadherin CP-14.81%**

**CP-14.81%	WT-89.92%**

**WT 0.26 %	CP-96.26%**

**WT- 89.92% CP-14.81%**

**E-cadherin CP-14.81%**

**E-cadherin WT-89.92%**

**Snail WT-3.24%**

**Snail CP-29.14%**

**WT- 3.24% CP-29.14%**

**Slug WT-2.78%**

**Slug CP-25.84%**

**WT-2.78% CP-25.84%**

**Goosecoid WT-3.74%**

**Goosecoid CP-43.73%**

**WT- 3.74% CP-43.73%**

**LEF1 WT-8.69%**

**LEF1 CP-55.22%**

**WT-8.69% CP-55.22%**

**Figure SF9: Flow cytometry analysis of neural and EMT markers in SKOV3WT and SKOV3CP cells.** Incubation of SKOV3^WT^ cells with cisplatin promoted neuron-like morphology and the overexpression of different proteins specific for neuronal and EMT conditions. In each case, the first two figures illustrate the individual expression of each marker in SKOV3^WT^ and SKOV3^CP^ cells and the third figure is the superposition of the expression of both the cells. Histograms in grey represent the isotype controls individually applied according the species. Representative histograms of n=3

**Figure SF10**: RBG decomposition of pictures from Figure 5, Panel D (main document)

OC236 Control

2Ab

2Ab

DAPI

OC236 Control

2Ab

2Ab

DAPI

OC236 Control

2Ab

2Ab

DAPI

OC236

EpCAM

Notch3

DAPI

OC236

EpCAM

PAX6

DAPI

OC236

EpCAM

PAX6

DAPI

OC236

EpCAM

PAX6

DAPI

OC236

PAX6

DAPI

OC236

PAX6

DAPI

Twin cell

OC236

EpCAM

PAX8

DAPI

OC236

EpCAM

PAX9

DAPI

OC236

EpCAM

Klf-4

DAPI

OC236

EpCAM

SOX2

DAPI

OC236

EpCAM

Slug

DAPI

OC236

EpCAM

Vimentin

DAPI

OC236

EpCAM

βIII-tubulin

DAPI

OC236

EpCAM

Doublecortin

DAPI

OC236

EpCAM

Stathmin

DAPI

OC236

EpCAM

ChgA

DAPI

OC236

EpCAM

PNMA1

DAPI

OC236

EpCAM

KRT80

DAPI

**Figure SF10**: RBG decomposition of pictures from **Figure 5, Panel D** (main document). In order to facilitate the subcellular distribution of different markers presented in **Figure 5** of the main document, we are providing the channel decomposition of the immunofluorescence experiments. In the ascites populations (OC236) there are islands of Vimentin positive/EpCAM negative cells, indicating that EMT is continuously occurring in vivo.

PAX6 is observed ectopically distributed, therefore not active, in the cytoplasm in massive form, although in the nucleoplasm region is also observable. Twin cells are highly expressing PAX6. In general, is also expressed in islands form in primary OC cell cultures (platin treated). The subcellular localization of PAX6 in the island is observable in both cytoplasm and nucleus. However, it is described that in some cell types PAX6 may be present in the cytoplasm (https://www.proteinatlas.org/ENSG00000007372-PAX6/subcellular). PAX8 expression is undistinguished from the cells forming island and surrounding cells. Its subcellular localization is found in both cytoplasm and nucleoplasm. Therefore difficult to argue that is characteristic for the island cells. Its expression is however massive with many aggregates in the cytoplasm. PAX9 expression is very distinguished in the cells that form islands in primary OC cell cultures (platin treated). Its subcellular localization is distributed along the cell body, although is observable an accumulation in the nucleoplasm.

The neurogenic locus notch homolog protein 3 (Notch3) is highly expressed in cells, which form the islands in OC primary cells. Those cells however do not express EpCAM, indicating this distorted relation observable in the case above. Notch3 powerfully contribute to stem cell maintenance and it is a master regulator of cell differentiation.

KRT80 is expressed by all cells in the OC236. Its cellular localization is in cytoplasm. PNMA1 is also expressed in all cell populations within OC236. It appears however, that the EpCAM^-^ nest cells express more than the EpCAM^+^ cells.

ChgA is highly expressed by EpCAM^-^ nest cells. However, the EpCAM^+^ cells although are losing the Chromogranin A, they retain detectable protein in their cytoplasm. This is highly relevant since we have confirmed the loss of neuronal markers towards the epithelization of cells in colorectal carcinomas. Thus, in much differentiated tumor tissues is less detectable but their origin are clearly neuronal. Stathmin is localized in both cell populations, although the EpCAM^-^ nest cells appear to express large quantities of this neural protein.

EpCAM

βIII-tubulin

DAPI

**SKOV3^WT^**

**Figure SF11**: **Simultaneous expression of EpCAM and βIII-tubulin in SKOV3^WT^**. The expression of βIII-tubulin in SKOV3^WT^ is restricted to few cells. The expression of EpCAM is widely found in SKOV3^WT^ cell populations. Of note, there are some cells with low levels of EpCAM and βIII-tubulin. Representative picture. White scale bar, 50 µm.

SKOV3^CP^

EpCAM

βIII-tubulin

DAPI

**Figure SF12**: Simultaneous expression of EpCAM and βIII-tubulin in SKOV3^CP^. The expression of βIII-tubulin in SKOV3^CP^ is observed in several cells, whereas the expression of EpCAM is very low or absent, indicating the phenotype conversion of cells. Representative picture. White scale bar, 50 µm.

SKOV3^CP^

EpCAM

Vimentin

DAPI

**Figure SF13**: Simultaneous expression of EpCAM and Vimentin in SKOV3^CP^. The expression of EpCAM in SKOV3^CP^ is very low or absent, whereas the expression of Vimentin is the most prominent marker observed, indicating the phenotype conversion of cells. Representative picture.

**Figure SF14**: Expression of some genes in ovarian carcinoma cells isolated from ascites of patients treated with platinum compounds applied with PIPAC. Data was compared with both wildtype OVCAR-3 and cisplatin exposure, as reference. Overexpression of β III-tubulin, HIF1A and Stathmin was observed. Thus, EMET via platin compounds could be associated with the current treatments of ovarian carcinomas.

HES1 in SKOV3^WT^

HES1 in OVCAR-3

**A**

HES1 in SKOV3

|  |  |
| --- | --- |
| Controls WT | 4805.25 |
| Controls DMSO | 5609.09 |
| DAPT 0,1 µM | 8382.88 |
| DAPT 1 µM | 3369.21 |
| DAPT 5 µM | 1918.44 |
| RG4733 0,1 µM | 1960.87 |
| RG4733 1 µM | 1466.17 |
| RG4733 5 µM | 1189.8 |

HES1 in OVCAR-3

|  |  |
| --- | --- |
| Controls WT | 2686.85 |
| Controls DMSO | 2698.19 |
| DAPT 0,1 µM | 1555.72 |
| DAPT 1 µM | 1142.98 |
| DAPT 5 µM | 1062.95 |
| RG4733 0,1 µM | 1057.95 |
| RG4733 1 µM | 854.983 |
| RG4733 5 µM | 792.116 |

**B**

SKOV3^CP^

Control

SKOV3^CP^

2 µg/mL CP

SKOV3^CP^

2 µg/mL CP

SKOV3^CP^

2 µg/mL CP

SKOV3^CP^

2 µg/mL CP +

1 µM RG-4733

SKOV3^CP^

2 µg/mL CP +

1 µM RG-4733

SKOV3^CP^

2 µg/mL CP +

1 µM RG-4733

SKOV3^CP^

2 µg/mL CP +

5 µM DAPT

SKOV3^CP^

Control

SKOV3^CP^

Control

SKOV3^CP^

2 µg/mL CP +

5 µM DAPT

SKOV3^CP^

2 µg/mL CP +

5 µM DAPT

**Figure SF15**: Inhibition of HES1 by γ-secretase inhibitors. Both SKOV3^WT^ and OVCAR-3 OC cells were incubated with γ-secretase inhibitors RG-4733 or DAPT, both indirect inhibitors of Notch pathway, for 120 hours. After this time, cell extracts were subjected to WB for the detection of HES1. A downregulation of HES1, which is governed by Notch TFs, is seen (**A**). However, this inhibition do not induce observable changes in the “arborization” of SKOV3^CP^ (**B**).
